# Discovery of Bacterial Key Genes from 16S rRNA-Seq Profiles That Are Associated with the Complications of SARS-CoV-2 Infections and Provide Therapeutic Indications

**DOI:** 10.3390/ph17040432

**Published:** 2024-03-28

**Authors:** Md. Kaderi Kibria, Md. Ahad Ali, Muhammad Yaseen, Imran Ahmad Khan, Mashooq Ahmad Bhat, Md. Ariful Islam, Rashidul Alam Mahumud, Md. Nurul Haque Mollah

**Affiliations:** 1 Bioinformatics Laboratory, Department of Statistics, University of Rajshahi, Rajshahi 6205, Bangladesh; kibria.stt@tch.hstu.ac.bd (M.K.K.); ahad.chembd@gmail.com (M.A.A.); ariful.stat.bio@gmail.com (M.A.I.); 2 Department of Statistics, Hajee Mohammad Danesh Science and Technology University, Dinajpur 5200, Bangladesh; 3 Department of Chemistry, University of Rajshahi, Rajshahi 6205, Bangladesh; 4 Institute of Chemical Sciences, University of Swat, Main Campus, Charbagh 19130, Pakistan; muhammadyaseen.my907@gmail.com; 5 Department of Chemistry, Government College University, Faisalabad 38000, Pakistan; imrankhan707470@gmail.com; 6 Department of Pharmaceutical Chemistry, College of Pharmacy, King Saud University, Riyadh 11421, Saudi Arabia; mabhat@ksu.edu.sa; 7 NHMRC Clinical Trials Centre, Faculty of Medicine and Health, The University of Sydney, Camperdown, NSW 2006, Australia; rashed.mahumud@sydney.edu.au

**Keywords:** SARS-CoV-2 infections, bacterial co-infections, 16S rRNA-seq profiles, bacterial key genes (bKGs), drug repurposing, bioinformatics analysis

## Abstract

SARS-CoV-2 infections, commonly referred to as COVID-19, remain a critical risk to both human life and global economies. Particularly, COVID-19 patients with weak immunity may suffer from different complications due to the bacterial co-infections/super-infections/secondary infections. Therefore, different variants of alternative antibacterial therapeutic agents are required to inhibit those infection-causing drug-resistant pathogenic bacteria. This study attempted to explore these bacterial pathogens and their inhibitors by using integrated statistical and bioinformatics approaches. By analyzing bacterial 16S rRNA sequence profiles, at first, we detected five bacterial genera and taxa (*Bacteroides*, *Parabacteroides*, *Prevotella Clostridium*, *Atopobium,* and *Peptostreptococcus)* based on differentially abundant bacteria between SARS-CoV-2 infection and control samples that are significantly enriched in 23 metabolic pathways. A total of 183 bacterial genes were found in the enriched pathways. Then, the top-ranked 10 bacterial genes (*accB*, *ftsB*, *glyQ*, *hldD*, *lpxC*, *lptD*, *mlaA*, *ppsA*, *ppc*, and *tamB*) were selected as the pathogenic bacterial key genes (bKGs) by their protein–protein interaction (PPI) network analysis. Then, we detected bKG-guided top-ranked eight drug molecules (Bemcentinib, Ledipasvir, Velpatasvir, Tirilazad, Acetyldigitoxin, Entreatinib, Digitoxin, and Elbasvir) by molecular docking. Finally, the binding stability of the top-ranked three drug molecules (Bemcentinib, Ledipasvir, and Velpatasvir) against three receptors (*hldD*, *mlaA,* and *lptD*) was investigated by computing their binding free energies with molecular dynamic (MD) simulation-based MM-PBSA techniques, respectively, and was found to be stable. Therefore, the findings of this study could be useful resources for developing a proper treatment plan against bacterial co-/super-/secondary-infection in SARS-CoV-2 infections.

## 1. Introduction

The novel coronavirus disease 2019 (COVID-19), one of the most severe respiratory infections caused by the Severe Acute Respiratory Syndrome CoronaVirus-2 virus (SARS-CoV-2), remains a risk factor for human life. As of 7 January 2024, there have been over 774 million cases and over seven million deaths worldwide [1]. Though different vaccination programs have been reducing the rate of infection, a significant number of people are still being infected around the globe due to the random mutation in the RNA sequence of SARS-CoV-2 [2], which demands effective drugs as supplementary treatment for COVID-19. The spike (S) protein of SARS-CoV-2 interacts with the host ACE2 (angiotensin-converting enzyme 2) protein to initiate the infection [3,4]. Inhibition of this protein is essential to neutralize SARS-CoV-2 before the start of a cytokine storm, for which the immune system generates a number of inflammatory signals that can cause organ failure and patient death. The major protease (MPro/3ClPro), the papain-like protease (PLpro) [5,6] and the RNA-dependent RNA polymerase (RdRp) [7,8] of SARS-CoV-2 are associated with the infections by catalyzing the replication of RNA from an RNA template. Therefore, these proteins were used as targets/receptors to explore candidate drugs against SARS-CoV-2 infections [6,9]. However, COVID-19 patients with weak immunity may suffer from different complications due to microbial co-infections/super-infections/secondary-infections [10,11,12,13,14,15,16]. It should be noted here that co-infections refer to the simultaneous infection with two or more different microbial pathogens [14,17]. Super-infection occurs when already infected patients are re-infected with other microbial pathogens [15,16]. Secondary infections occur when already infected patients are re-infected, depending on the outcomes of the first infection [14,17,18]. COVID-19 patients often suffer from different symptoms, including fever [19], cough [20], sore throat [21], congestion or runny nose [22], difficulty breathing [23], loss of smell or taste [24], fatigue [21], muscle pain [25], headache [26], nausea or vomiting [27], diarrhea [28], meningitis [29], pneumonia [29] that are associated with different microbial pathogens [30,31,32,33]. Therefore, microbial pathogens may be considered the critical risk factors for different complications and the increasing mortality rates of COVID-19 patients [14,15,16,17,34]. In order to inhibit microbial pathogens, it is required to identify pathogenic genes that may lead to exploring potential inhibitors. It may be mentioned here that either disease-causing microbial proteins or host-proteins are considered the receptor proteins for exploring candidate drug molecules in order to inhabit microbial infections [6,9,35,36,37,38,39,40], since pathogenic proteins interact with host-proteins to develop infections [39,41,42].

There are some studies based on 16S rRNA-Seq profiles that have explored the association of microbial compositions with the complications of SARS-CoV-2 infections [43,44,45,46,47,48,49,50,51,52,53,54,55]. For instance, the bacterial composition of the respiratory tract and gut is associated with the occurrence and severity of disease in numerous respiratory viral infections (RVs) affecting the eventual respiratory health of COVID-19 [43]. The bacterial phyla groups *Firmicutes* and *Bacteroidetes,* as well as commensals of the phyla, are directly associated with the pathogenesis and severity of SARS-CoV-2 infections due to their regulatory function with the ACE2 host gene [53]. The *Bacteroidetes* phylum is associated with the downregulation of the ACE2 gene, while the *Firmicutes* phylum is associated with the upregulation, resulting in lower and higher infection rates, respectively [54]. A study has analyzed the oral and gut microbiome data and found that *Rothia mucilaginosa* and *Granulicatella* bacterial taxa are related to SARS-CoV-2 infections [55]. Thus, we observed that previous studies based on 16S rRNA-Seq profiles have explored only the bacterial taxa that are associated with co-/super-/secondary infections. They did not explore such infection-causing bacterial genes and their inhibitors. Two studies recommended antimicrobial agents in order to inhibit microbial pathogens. However, some microbial pathogens may progressively develop resistance to antimicrobial drugs due to environmental changes. This type of drug-resistant microbial pathogens is gradually increasing due to the availability of a small number of antimicrobial agents [56,57]. In this case, multi-targeted different variants of alternative antimicrobial drugs may be essential for effective treatment against co-/super-/secondary infections. Therefore, this study attempted to explore such infection-causing bacterial key genes (bKGs) based on 16S rRNA-Seq profiles and associated drug molecules in order to inhibit those infection-causing pathogenic bacteria, since 16S rRNA-Seq profile analysis performs better in order to identify novel pathogens and non-cultured bacteria [58,59].

## 2. Results

### 2.1. Diversity Analysis

**Figure 1:**
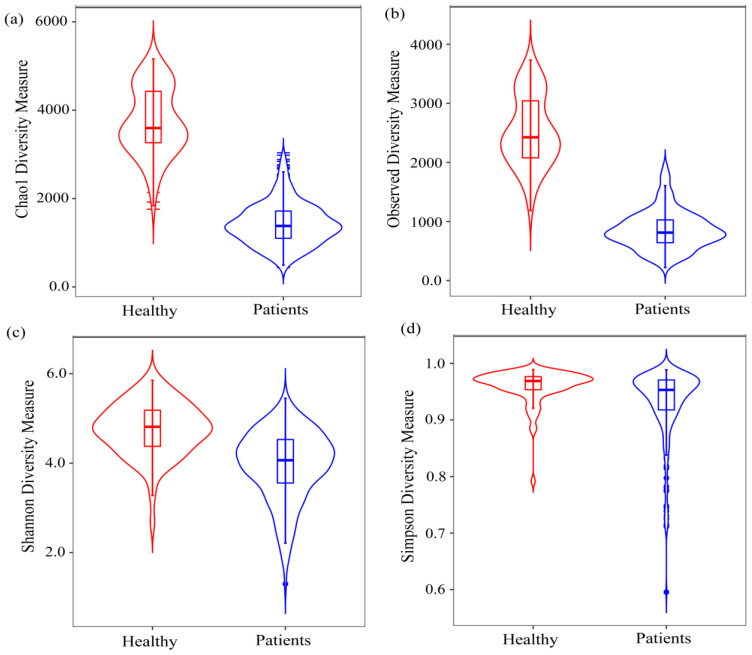
Violin plot for alpha-diversity results. These results demonstrate the differences in bacterial community between COVID-19 patients and healthy samples based on the (**a**) Chao1, (**b**) observed, (**c**) Shannon, and (**d**) Simpson index. The Wilcoxon signed-rank test was used to test the significance (*p*-value < 0.05) difference between patients and healthy individuals.

All alpha diversity indices (richness measure: observed and Chao1, and evenness measure: Shannon and Simpson) of the oral and gut microbiota showed a significant difference (*p* < 0.01) between the COVID-19 patients and healthy group (Figure 1). The median observed index was 2941.5 (IQR: 2502, 3874) for the healthy group and 937.5 (IQR: 717.25, 1199.75) for the COVID-19 patients, indicating a significant decrease in observed species. Similarly, the median of Chao1 index was 4852.912 (IQR: 4250.33, 6318.36) for healthy patients and 1729.328 (IQR: 1303.84, 2200.93) for COVID-19 patients. The Shannon index was 4.875 (IQR: 4.455, 5.263) and 4.145 (IQR: 3.605, 4.556), and the Simpson index was 0.969 (IQR: 0.954, 0.977) for healthy and 0.953 (IQR: 0.919, 0.971) for COVID-19 patients, respectively (see Table S1 in Supplementary Materials). The findings indicate a significant decrease in four alpha diversity species among COVID-19 patients compared to healthy individuals, with a substantial effect size observed in Cliff’s Delta statistics for observed, Chao1, and Shannon indices, highlighting a significant distinction between the COVID-19 and healthy groups (see Table S1 in Supplementary Materials).

**Figure 2:**
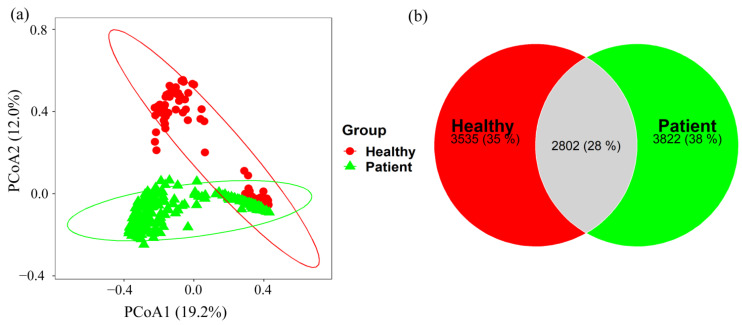
(**a**) Bacterial genetic diversity between COVID-19 and control samples was investigated by principal coordinates analysis (PCoA) with the beta diversity indices, where healthy and patient samples were represented by dot and a triangle symbols, respectively. (**b**) Venn diagrams showed the percentage of shared OTUs between COVID-19 patients and healthy controls.

In addition to alpha diversity indices, beta diversity was employed to evaluate compositional changes in the microbial communities. Principle Coordinate Analysis (PCoA) based on Bray–Curtis distance revealed distinct clustering patterns among samples with varied distributions (see Figure 2a). Permutational Multivariate Analysis of Variance (PERMANOVA) on Bray–Curtis distance (F = 22.575, *p*-value: 0.001) indicated a significant difference between COVID-19 patients and healthy individuals.

A total of 2802 OTUs were found to be shared between COVID-19 patients and healthy controls, accounting for 37.71% and 32.60% of the total OTUs in healthy controls and COVID-19 patients, respectively (see Figure 2b). These results suggest that compositional differences were associated with the presence or absence of specific taxa and their relative abundances in the samples.

### 2.2. Taxonomy Analysis and Identification of Differentially Abundant Bacterial Compositions (DABCs)

**Figure 3:**
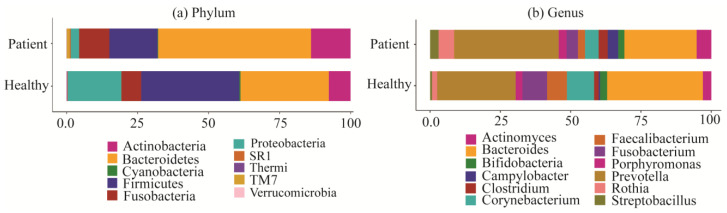
Composition of bacterial abundances at the phylum and genus levels across COVID-19 and control samples. (**a**) Histograms of relative abundances of bacteria for the 10 most abundant bacterial phyla across COVID-19 and control samples. (**b**) Histogram of relative abundance of bacteria for the 12 most abundant bacterial genera across COVID-19 and control samples.

The oral and gut microbiota profiles at the phylum and genus levels consisted of approximately 22 phyla and 210 genera, respectively. Among them, the top 10 most abundant phyla and the top 12 most abundant genera are shown in Figure 3. The results indicate that the abundance of the *Bacteroidetes* phylum was relatively higher in COVID-19 patients than in healthy subjects (26.9% vs. 19.6%), followed by *Actinobacteria* (13.941% vs. 7.703%), *Fusobacteria* (10.66% vs. 6.92%), *TM7* (1.037% vs. 0.011%), and *SR1* (0.382% vs. 0.122%). On the other hand, the relative abundances of *Firmicutes*, *Proteobacteria*, *Cyanobacteria*, and *Verrucomicrobia* phyla decreased in COVID-19 patients compared to healthy subjects. However, *Thermi* exhibited less significant changes in patients and healthy individuals at the phylum level (Figure 3a and Table S2 in Supplementary Materials). Also, among the 12 most abundant genera in the oral and gut microbiota in COVID-19 patients, *Prevotella* (37.21% vs. 27.85%), *Rothia* (5.49% vs. 1.84%), *Actinomyces* (5.17% vs. 2.95%), *Clostridium* (3.15% vs. 1.59%), *Streptobacillus* (3.06% vs. 0.68%), and *Campylobacter* (3.70% vs. 0.51%) showed increased relative abindances in COVID-19 patients compared to healthy subjects. Conversely, *Bacteroides* (25.73% vs. 34.08%), *Corynebacterium* (4.89% vs. 9.62%), *Fusobacterium* (4.02% vs. 8.82%), and *Faecalibacterium* (2.48% vs. 7.05%) were comparatively less abundant in COVID-19 patients than in healthy individuals (Figure 3b and Table S2 in Supplementary Materials). Alternatively, insignificant changes were observed in the cases of *Porphyromonas* and *Bifidobacterium*.

**Figure 4:**
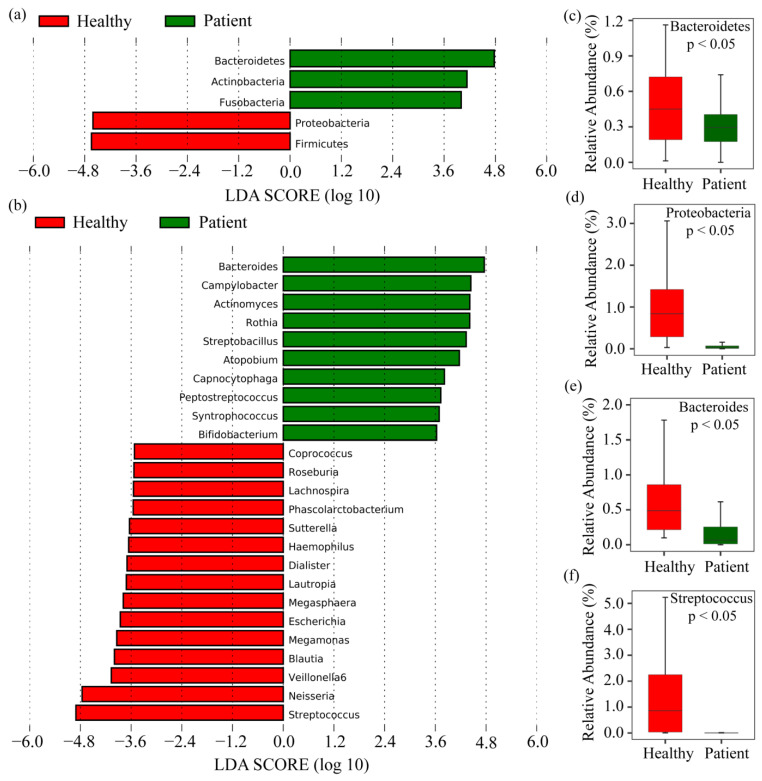
Identify differentially abundant bacteria between COVID-19 and control samples at the phylum and genus levels. (**a**) Histogram of LDA scores to display the significantly differentially abundant phyla between two groups (LDA score > 3.5 and *p* < 0.05). (**b**) Histogram of LDA scores to display the significantly differentially abundant genus between two groups (LDA score > 3.5 and *p* < 0.05). (**c,e**) The relative bacterial abundance for two groups of two significant phyla levels at Bacteroidetes and Bacteroides was displayed by using a Box plot. (**d,f**) The relative bacterial abundance of two significant genus levels at Proteobacteria and Streptococcus for two groups was displayed by using a box plot.

Based on their relative abundances, it is possible to identify which phyla and genera were more abundant, but it remains unclear which taxa were significantly differentially abundant in the SARS-CoV-2 infected group. LEfSe analysis was used to identify significantly abundant phyla and genera between groups (LDA score > 3.5, *p* < 0.05). The results reveal that five phyla were significantly different between the groups. Among them, *Bacteroidetes*, *Actinobacteria*, and *Fusobacteria* phyla were significantly enriched in COVID-19 patients, while *Proteobacteria* and *Firmicutes* were significantly decreased (see Figure 4). On the other hand, 25 genera showed different abundance between the groups, whereas the *Bacteroides*, *Campylobacter*, *Actinomyces,* and *Rothia* genera were highly enriched in COVID-19 patients, while *Streptococcus*, *Neisseria Veillonella*, *Blautia*, *Megamonas Escherichia,* and *Megasphaera* were less enriched compared to healthy patients. The LDA scores revealed a significant abundance of *Bacteroides* and *Streptococcus* as microbiological biomarkers in the treatment group and healthy group (see Figure 4). These results demonstrate a notable difference in gut microbiota composition between healthy and COVID-19 patients.

Further, the zero-inflated Gaussian (ZIG) model was employed using the metagenomeSeq R package to identify significantly differential bacterial genera based on the adjusted *p*-value and log2FC values. Almost 111 bacterial genera showed significant differences between the groups at *p* < 0.05, which were used for further analysis. All the genera identified as differential in the LEfSe analysis were included among the 111 differentially abundant genera identified in the ZIG model analysis. We represented the top 20 highly differential genera based on the adjusted *p*-value and log2FC in Table 1, noting that nine bacterial genera were upregulated while the remaining 11 were downregulated in COVID-19 patients. Particularly, genera such as, *Neisseria*, *Streptococcus*, *Leptotrichia,* and *Veillonella* were significantly decreased in COVID-19 patients, whereas *Bacteroides*, *Parabacteroides*, *Prevotella,* and *Clostridium* were significantly increased compared to healthy individuals. Consequently, in COVID-19 infections, the abundances of genera such as *Bacteroides*, *Parabacteroides*, *Prevotella Clostridium*, *Atopobium,* and *Peptostreptococcus* showed significant increases.

### 2.3. Identification of Pathway-Based Bacterial Key Genes (bKGs) from DABCs

**Figure 5:**
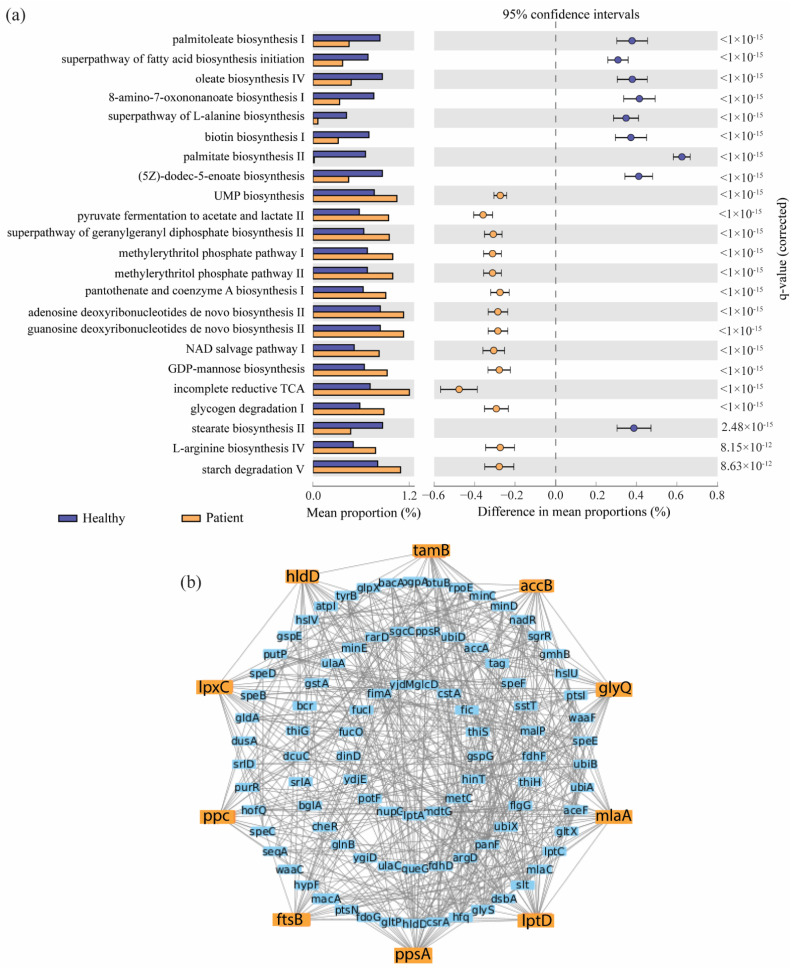
Identifying metabolic pathways and associated functional key genes that were associated with SARS-CoV-2 infection. (**a**) Prediction of metabolic pathway contents correlated with COVID-19 (patients) and control (healthy) groups. Extended error bar-plot representing the differences in mean proportions for each pair of groups with each pathway. Welch’s t statistic produced q-values for the two-tailed test, which were adjusted by controlling FDR at 0.05 with the Benjamini–Hochberg method. (**b**) Protein–protein interaction networking to select bKGs, where the orange color indicates bKGs.

To comprehend the functional role of COVID-19-related bacteria, PICRUSt2 analysis was conducted to predict gut microbiota metagenomes from the 111 differentially abundant genera identified in the ZIG model. Furthermore, the results of this functional analysis were compared to the Kyoto Encyclopedia of Genes and Genomes (KEGG) orthologs to assess differences in predicted functional gene abundances. As a result, 23 MetaCyc signaling pathways differed significantly in the mean proportions between the COVID-19 and healthy groups (see Figure 5a). Of these, 14 MetaCyc pathways showed significant increases, including incomplete reductive TCA and pyruvate fermentation to acetate and lactate II, while 9 pathways, including palmitate biosynthesis II, palmitoleate biosynthesis I, and oleate biosynthesis IV, were significantly decreased in the COVID-19 patients group. Furthermore, we identified 183 KEGG genes corresponding to 23 differentially abundant pathways and hence explored the 10 most functional bKGs *(accB*, *ftsB*, *glyQ*, *hldD*, *lpxC*, *lptD*, *mlaA*, *ppsA*, *ppc*, and *tamB)* through protein–protein interaction network analysis. These identified bKGs may represent possible functional gene contents altered due to secondary infection with SARS-CoV-2, and the findings were used for further molecular docking analyses (see Figure 5b).

### 2.4. bKGs-Guided Drug Repurposing by Molecular Docking

**Figure 6:**
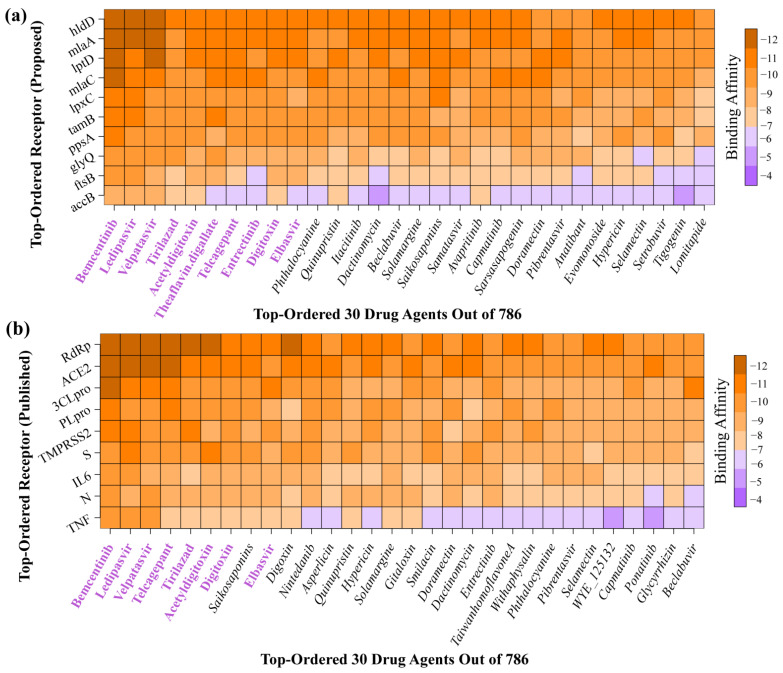
The protein–ligand binding affinity score matrices were displayed, where the X-axis represents the top-ranked 30 drug molecules and the y-axis represents the corresponding ordered bKGs. (**a**) The image of the score matrix indicates the binding affinities between the proposed 10 bKGs/ receptors and the 30 top-ranked drug molecules. The top 10 drug molecules, determined by their binding affinity scores, are highlighted in purple, and (**b**) the score matrix indicates the binding affinities between the COVID-19-causing top-ranked 9 published key receptors and the 30 top-ranked drug molecules with 8 drugs are being common with the proposed top 10 drug indicated by the purple color.

To select the top-ranked potential candidate drug molecules for COVID-19 treatment from the pools of 786 published molecules using molecular docking, we considered our proposed top-ranked 10 bKG-mediated proteins as receptors. Subsequently, we downloaded the 3D structures of our key proteins *accB*, *ftsB*, *glyQ*, *hldD*, *lpxC*, *lptD*, *mlaA*, *ppsA*, *ppc*, and *tamB* from the Protein Data Bank (PDB), corresponding to the source codes *4hr7*, *6h9o*, *7qcf*, *4ej0*, *4isa*, *4rhb*, *5nuq*, *7akc*, *1fiy*, and *5vtg*, respectively. Heteroatoms, water molecules, and unused ligands were then removed using BIOVIA Discovery Studio and PyMOL. Subsequently, protein energy minimization was conducted using the GROMOS 43B1 force field. Finally, each protein was converted into an acceptable PDBQT format using AutoDock Tools. Molecular docking analyses were performed between the proposed 10 receptors and the 786 meta-drug agents to calculate the binding affinity scores (kcal/mol) for each receptor–ligand pair. These binding affinity scores were arranged in a matrix A = (A_ij_) in descending order, with receptor proteins in rows and ligands in columns (see Figure 6a). Consequently, we selected the top ranked 10 drugs (Bemcentinib, Ledipasvir, Velpatasvir, Tirilazad, Acetyldigitoxin, Theaflavin digallate, Telcagepant, Entreatinib, Digitoxin, and Elbasvir) as candidate drug molecules, with an average binding affinity score of −9.618 kcal/mol against the proposed 10 receptors. To evaluate the binding performance of our proposed 10 candidate drug molecules against the top-ranked independent receptors, we reviewed 98 published articles identifying hub proteases/proteins with SARS-CoV-2 infections. This review yielded a total of 215 hub proteins (see Table S4 in Supplementary Materials).

Out of 215, only 9 hub proteins (*ACE2*, *RdRp*, *3CLpro*, *S*, *TMPRSS2*, *PLpro*, *IL6*, *TNF*, and *N*) were found in at least six articles. These hub proteins were considered independent receptors to investigate their binding capacity with our proposed drug molecules. We downloaded those independent receptor proteins from the Protein Data Bank (PDB) with source codes *2ajf*, *7bv2*, *6lu7*, *6vsb*, *7meq*, *6w9c*, *1alu*, *1a8m*, and *6m3m*, respectively. Then, molecular docking analysis was performed with these nine independent hub proteins against the same set of published 786 ligands. Then, their binding affinities were arranged in descending order and visualized in a matrix plot (see Figure 6b).

**Figure 7:**
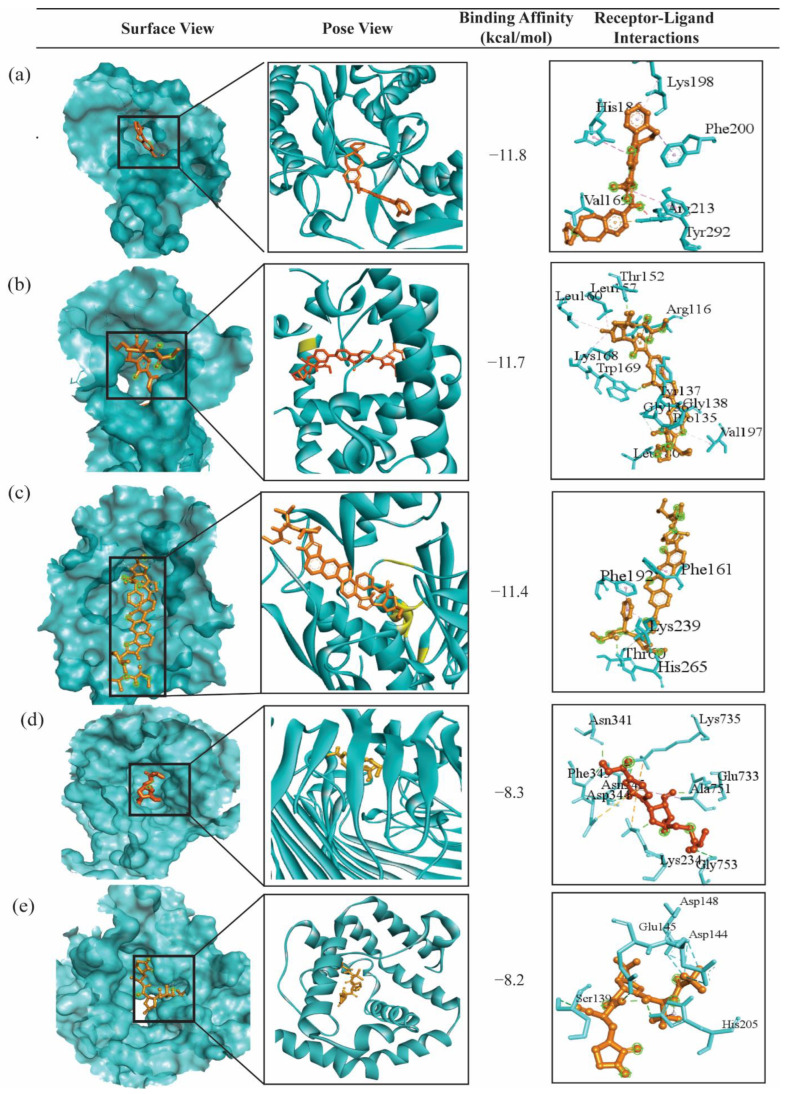
Visualization of top-scorer target-ligand complexes, with lightseagreen color representing the protein and gold indicating the ligand. The interacting complex (3D) surface view, pose view, and interactions were shown in the 1st, 2nd, and 3rd columns, respectively. (**a**) Overall top-ranked complex between Bemcentinib and hldD. (**b**) Overall 2nd top-ranked complex between Ledipasvir and mlaA. (**c**) Overall 3rd top-ranked complex Velpatasvir and lptD. (**d**) Complex between lptD and Molnupiravir, which is the highest scorer complex with Molnupiravir only. (**e**) Complex between mlaA and the Nirmatrelvir drug, which is the highest scorer complex with Nirmatrelvir only. Here, Ledipasvir, Molnupiravir, and Nirmatrelvir are three FDA-approved COVID-19 drugs. The last two complexes are displayed here to compare them with the overall top-scoring three proposed complexes (the first three).

The top-ranked 10 drug molecules produced a binding affinity score lower than −7.0 kcal/mol against the top-ranked 9 independent receptors. Among the top 10 ligands (according to the binding affinity), 8 of them (Bemcentinib, Ledipasvir, Velpatasvir, Tirilazad, Acetyldigitoxin, Telcagepant, Digitoxin, and Elbasvir) were common with our proposed 10 lead compounds (see Figure 6). Therefore, we considered these 8 drug molecules as the proposed candidates. Interestingly, three ligands (Bemcentinib, Ledipasvir, and Velpatasvir) were ranked highest against both proposed and independent receptors, suggesting their potential effectiveness against COVID-19. To assess the binding performance of our proposed drug molecules compared to the FDA approved two COVID-19 drugs (Molnupiravir and Nirmatrelvir) [60,61], we analyzed their overall docking scores against both proposed and independent receptors (see Table S5 in Supplementary Materials). Interestingly, neither Molnupiravir nor Nirmatrelvir ranked among the top 30 drug molecules based on their average binding affinity scores in both scenarios (see Figure 6). The interacting properties of these five target-ligand complexes are displayed in Figure 7 and also given in Table S6. The interacting complex (3D) surface view, pose view, and protein–ligand interactions are shown in Figure 7. The hldD-Bemcentinib complex was formed with one Pi-donor hydrogen bond at ARG213, one Pi-sigma bond at PHE200, two Pi-Pi T-shaped bonds at HIS186 and TYR292, an alkyl bond at VAL168, and a Pi-Alkyl bond at LYS198. Similarly, the other 4 target-ligand complexes were formed as given by their data in Table S6.

Additionally, the effectiveness and indemnity level of the top three lead compounds were assessed by evaluating their toxicological and pharmacokinetic properties (see Table S3 in Supplementary Materials). Central nervous system (CNS) permeability is less than −2, which indicates an inability to permeate the blood–brain barrier. Moreover, the number of H-bond acceptors and donors for the three compounds was less than ten and five, respectively, suggesting that the compounds are plausible drug candidates. Finally, no toxicity or carcinogenic profiles were observed for our three lead compounds (see Table S3 in Supplementary Materials).

**Figure 8:**
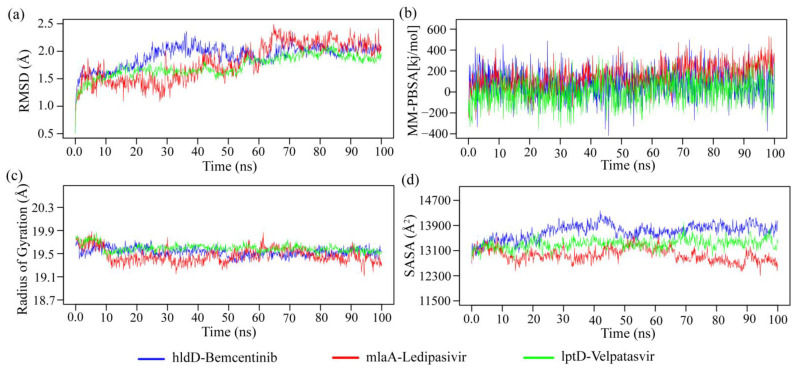
The molecular dynamic simulation of the proposed three docked complexes. (**a**) root mean square deviation of the alpha carbon atoms, (**b**) binding free energy of the complexes, where a more positive value indicates better binding, (**c**) degree of rigidity and compactness analysis of the complexes, and (**d**) protein volume with expansion analysis.

A molecular dynamics (MD) simulation was conducted to verify the structural stability of the top three target-ligand complexes obtained from docking. The root mean square deviations (RMSD) of C-alpha atoms for the docked complexes are shown in Figure 8a. At the beginning of the simulation, all three complexes, hldD-Bemcentinib, mlaA-Ledipasvir, and lptD-Velpatasvir showed the same trend. However, the hldD-Bemcentinib complex exhibited a higher flexibility at 20–40 ns, while the mlaA-Ledipasivir complex showed a lower trend after 20 ns until 30 ns. Eventually, all three complexes reached a steady state after 60 ns throughout the simulation period. The RMSD value of the three complexes was less than 2.5 Å, indicating their stable and rigid structure. Furthermore, the stable and steady nature of the three complexes was assessed using the radius of gyration (Rg) and the solvent accessible surface area (SASA) (see Figure 8c,d). The lptD-velpatasvir complex exhibited a higher Rg, suggesting greater flexibility, while the mlaA-ledipasvir complex showed a lower Rg, indicating a more stable profile. On the other hand, the SASA analysis revealed changes in protein volume over time (see Figure 8d), with a lower SASA value indicating a condensed complex and a higher SASA value indicating an extended surface area.

The SASA values for the hldD-bemcentinib complex peaked at 25–50 ns, suggesting an expanded surface area, whereas the mlaA-ledipasvir complex exhibited lower SASA values at 15–45 ns, indicating a condensed surface. Conversely, the lptD-velpatasvir complex maintained a consistent profile throughout the simulation period. By 70 ns, all three complexes demonstrated stable and steady behavior. The binding energy of the three complexes was also calculated using the MM-PBSA method, with the more positive energy of the complexes indicating better binding (see Figure 8b). The mean binding energies of the hldD-bemcentinib, mlaA-ledipasvir, and lptD-velpatasvir complexes were 79.395, 148.595, and 12.078 kJ/mol, respectively. The mlaA-ledipasvir complex exhibited higher binding energy than the other two, suggesting better binding of the ligand molecules. The other two complexes had similar positive free energies, indicating better binding with these ligand molecules.

## 3. Discussion

Although SARS-CoV-2 is the main cause of COVID-19, there are some microbial compositions that are associated with the complications of COVID-19 by their co-infections, super-infections and secondary infections [14,15,16,17,34]. This study aims to explore co-/super-/secondary infections causing bacterial key genes (bKGs) and associated drug molecules through bioinformatics analysis. Alpha and beta diversity analyses showed that there are some bacterial compositions that are able to differentiate COVID-19 patients from healthy individuals (see Figure 3 and Figure 4). Bacterial compositions were observed at the phylum and genus level, revealing that at the phylum level, *Bacteroidetes* had a higher abundance in COVID-19 patients compared to healthy individuals. On the other hand, *Firmicutes*, *Proteobacteria,* and *Cyanobacteria* showed lower abundances in the gut microbiota of COVID patients than healthy patients. The ratio of *Firmicutes* to *Bacteroidetes* is known to influence health status [62], and an abundance of *Bacteroidetes* is also associated with acute diarrheal disease [63]. Also, the most abundant genera in COVID-19 patients compared to healthy individuals were *Prevotella*, *othia*, *Bacteroides,* and *Campylobacter* indicating higher abundances of these genera in infected individuals. While *Prevotella* has been associated with HIV in children and chronic obstructive pulmonary disease [64,65], Prevotella melaninogenica pathogen infection is also associated with meningitis diseases [66]. *Bacteroidetes* is also associated with gastric carcinogenesis [67,68] as well as colorectal cancer [69], whereas *Bacteroides Acidifaciens,* another differentially abundant bacterium, is implicated in liver damage [33]. *Campylobacter* enrichment in the gut microbiome of the patients is associated with diarrhea [28], gastrointestinal issues [70], and inflammatory bowel disease [71], while *Campylobacter fetus* pathogens can cause liver disease and diabetes mellitus [32]. Another upregulated genera identified in this study is *Clostridium difficile*, which is associated with diarrheal disease, another symptom of COVID-19 [72]. Alternatively, the genera *Neisseria*, *Streptococcus*, *Fusobacterium*, *Faecalibacterium*, *Corynebacterium*, and *Staphylococcus* were comparatively less abundant in COVID patients, suggesting that their reduced abundance may indicate COVID-19 infection. Among them, *Neisseria* contains two pathogens, *Neisseria gonorrhoeae* and *Neisseria meningitides,* which are associated with the meningococcal disease [73]. In another study, *Streptococcus* and *Fusobacterium* are often highly abundant in the oral cavity [74], and *Fusobacterium* is also highly associated with periodontal disease [75]. Another downregulated genus, *Haemophilus influenzae*, is implicated in meningitis and pneumonia diseases, which are common co-infections in COVID-19 patients [76]. On the other hand, the abundance of *Faecalibacterium in the* gut microbiome produces short-chain fatty acids, which have been associated with reduced intestinal inflammation [77]. Differentially abundant analysis revealed highly differential genera *Neisseria*, *Streptococcus*, *Leptotrichia,* and *Bacteroides* among the groups, belonging to the phyla *Proteobacteria*, *Firmicutes*, *Fusobacteria,* and *Bacteroidetes,* respectively.

We identified 23 significantly enriched metabolic pathways, including the incomplete reductive TCA and pyruvate fermentation to acetate and lactate II pathways, which were highly increased in COVID-19 patients, consistent with another study [78]. Although the *pyruvate fermentation* pathway does not release any ammonia, it does release butyrate, which can be harmful in conditions like chronic periodontitis [79]. On the other hand, *palmitate biosynthesis II*, *palmitoleate biosynthesis I*, and *oleate biosynthesis IV* significantly decreased COVID-19 infections. Some studies have shown that palmitate is essential in maintaining gut barrier integrity by controlling MUC2 secretion and function [80]. Another recent study found that *palmitate* increases the production of MUC2 in the goblet cells of the gut, leading to the formation of a thick mucus gel and maintaining the integrity of the gut barrier [81]. In addition to identifying metabolic pathways, we have identified the top 10 bacterial genes (*accB*, *ftsB*, *glyQ*, *hldD*, *lpxC*, *lptD*, *mlaA*, *ppsA*, *ppc*, and *tamB*) as bacterial key genes (bKGs) capable of facilitating secondary infection with SARS-CoV-2 through protein–protein interaction network analysis. Previous studies have highlighted the importance of certain genes in lipid metabolism, such as accB, which is significantly associated with diabetes and obesity in the Pakistani population [82]. On the other hand, another study explored that ppc genes are significantly associated with the metabolic disease hyperproteinemia, which is also associated with COVID-19 and increases the severity and mortality rate [83,84].

To explore the potential drug molecules, we conducted molecular docking corresponding to the identified bKGs. We selected the eight top-ranked ligands (bemcentinib, ledipasvir, velpatasvir, tirilazad, acetyldigitoxin, entreatinib, digitoxin, and elbasvir) based on their binding affinity scores, which were also supported by the independent receptors. Of the eight proposed drug molecules, only Ledipasvir is FDA-approved for COVID-19 [85]. Bemcentinib is FDA-approved for acute myeloid leukemia and passed clinical phase 2 trials for COVID-19 [86]. Velpatasvir is FDA-approved for the Hepatitis C virus (HCV) [87] and is under clinical trial for COVID-19. Tirilazad is an FDA-approved drug for brain cancer and spinal cord injury [88] and is under clinical trial for COVID-19. Acetyldigitoxin is used for the treatment of heart failure (HF) and is passing clinical trial phases for COVID-19. Digitoxin is passing a clinical phase 1 trial for COVID-19 [9]. Telcagepant is in a clinical phase 3 trial for acute migraine [89], but it is not yet under the clinical phase for COVID-19. Elbasvir is an FDA-approved drug for the treatment of hepatitis C virus genotype 1b (HCV GT1b) patients [90], and it is supported for COVID-19 by other studies [91], but is not yet considered for a clinical trial for COVID-19. To compare our suggested eight drug molecules against the two FDA-approved drugs (Molnupiravir and Nirmatrelvir) for COVID-19, we investigated their docking scores (kcal/mol) with our proposed and top-ranked published target proteins and found that our proposed drug molecules produced higher binding affinity scores in both cases. Interestingly, the top three drug molecules were the same against our proposed and published targets, and hence, we can conclude that these top three drug molecules, bemcentinib, ledipasvir, and velpatasvir, might be the most effective ligands for the treatment of COVID-19 infections. A study demonstrated that bemcentinib is efficacious in decreasing viral infection in lung cells, has a significant impact on SARS-CoV2 infection [92], and is also more effective in cancer treatment [93]. This drug molecule has no toxicity in the human body and is safe for treatment [92]. It prevents SARS-CoV-2 infections, as shown by viral transcripts in RNAseq studies and viral load in qRT-PCR studies of human lung epithelium, Vero-E6, and A549-hACE2 cells [94]. It reduces viral internalization but has no impact on viral binding. Ledipasvir is an approved drug for both COVID-19 and hepatitis C virus treatment with a good safety profile [95,96]. The antiviral activity of ledipasvir was identified in a cell-based screening assay against SARS-CoV-2 [97,98]. A study showed that the velpatasvir molecule produced the best binding affinity score against COVID-19 protease, which is also more effective against the hepatitis C virus treatment [99]. Furthermore, toxicity testing using the ADMET approach showed that the three complexes are the least toxic and the safest possible drugs for the treatment of SARS-CoV-2 infection. In addition, molecular dynamics (MD) simulation studies confirmed the structural stability of drug–target complexes by the RMSD, binding free energy, radius of gyration, and SASA parameters. So, our proposed drug molecules may have potential for the treatment of bacterial co-/super-/secondary infections with SARS-CoV-2 infections.

## 4. Limitations and Commercial Applications

This study was entirely computational-based. Therefore, the outputs of this study, including co-/secondary infection-causing bacterial key genes (bKGs) and candidate therapeutic agents, require experimental validation in a wet lab before going into the commercial production of effective drugs in order to inhibit bacterial pathogens for COVID-19.

## 5. Materials and Methods

### 5.1. Data Source and Description

In this study, we considered bacterial 16S rRNA sequence profiles and metadata on drug molecules that are associated with COVID-19, as described below.

#### 5.1.1. Collection of 16S rRNA Sequence Data

Bacterial 16S rRNA sequence profiles and associated metadata were downloaded from the NCBI online database with the bio-project number PRJNA684070. These samples were collected from the oral and gut microbiome signatures of hospitalized patients with COVID-19 and healthy individuals in Guangdong, China [55]. The dataset consisted of 297 samples, including 76 healthy and 221 COVID-19 patients. Previously, this dataset was analyzed to explore the bacterial taxa associated with SARS-CoV-2 infections for oral and gut samples separately [55]. In this study, we analyzed oral and gut samples jointly to explore common bacterial taxa/groups and their key genes that are associated with the severity of SARS-CoV-2 infections.

#### 5.1.2. Collection of Metadata on Drug Molecules

To explore bacterial key genes (bKGs) guided candidate drug molecules by molecular docking simulation for the treatment against bacterial co-/super-/secondary infections with SARS-CoV-2 infections, we collected 786 meta-drug agents from different published articles on COVID-19 (see Table S4 in Supplementary Materials).

### 5.2. Statistics and Bioinformatics Analysis

#### 5.2.1. Preprocessing of 16S rRNA Sequence Profiles

At first, we checked the quality of the raw sequence reads using FASTQC and then filtered the poor-quality reads using Trimmomatic-0.39 [100] with default parameters. After trimming the poor-quality reads, on average, almost 97% of the sequence reads survived for further study. Then, NGmerge (v0.2) was used to combine the overlapping areas between the paired-end trimmed reads with a minimum 5 bp overlap and a maximum of 10% mismatches [101]. Finally, the high-quality integrated reads were clustered into operational taxonomic units (OTUs), defined at 97% sequence similarity compared to the Greengenes reference database via Qiime2 software (version: 2023.9). A total of 12,671,011 OTUs with 42,663.33 mean numbers of OTUs per sample were obtained. For a large number of OTUs, filter the OTUs table with the minimum number of 5 samples that a feature observes. The filtered table contained 12,288,422 OTUs with a mean number of 41,797.35 (range: 80937-1778) OTUs per sample. Then, the OTU table was reduced to the lowest value of the total number of individuals observed in all samples.

#### 5.2.2. Diversity Analysis

The bacterial diversities within samples were investigated by using alpha diversity, measured by using four alpha diversity indices: observed, Chao1, Shannon, and Simpson [102], determined using phyloseq [103] R-package after trimming the minimum counts of all samples. The Chao1 and observed species are used to measure bacterial richness, while Shannon and Simpson quantitative indices measure species richness and evenness [104]. The significant differences across samples were tested by the nonparametric Wilcoxon sign rank test and the results were visualized using the ggbox R package [105]. To assess differences in species complexity between samples, beta diversity was used and measured using the Bray–Curtis distance based on normalization and log_10_ transformed rarefied OTU abundances [106]. In the R package, MicrobiotaProcess was used to perform principal coordinate analysis (PCoA) to obtain principal coordinates [107]. The variations in bacterial composition between the COVID-19 and healthy groups were investigated using PERMANOVA with Bray–Curtis distance through the vegan R-package with permutations set to 999 [108].

#### 5.2.3. Taxonomy Analysis and Identification of Differentially Abundant Bacterial Compositions (DABCs)

The Ribosomal Database Project (RDP Version 2.10) classifier was used to assign each representative sequence of OTU-clusters to the bacterial taxa in the Greengenes database (version: 13_5) by using Qiime2 (version: 2023.9) [109]. While the RDP classifier is based on the naïve Bayes model, which assigns a sequence to the closest match using a posterior score, we used linear discriminant analysis (LDA) effect size (LEfSe) on the Galaxy platform to identify the intergroup discriminant bacterial features at phylum and genus level across the patient groups (*p* < 0.05, LDA score > 3.5) “http://huttenhowersph.harva rd.edu/galaxy (accessed on 5 September 2023)” [110]. LEfSe uses the two-tailed nonparametric Kruskal–Wallis test to evaluate the significance of differences in OTUs in case–control groups. Additionally, a zero-inflated Gaussian mixture (ZIGM) model of mean group frequencies was applied to normalized count data to detect differentially abundant features or genera between case–control groups at a fixed *p*-value (*p* < 0.05) [111]. Bacterial count data were aggregated to the genus level, and abundant genera (>30 normalized counts per sample) were used for the ZIGM model. This analysis was performed using the R software package, version 4.3.3. The explored differential genera’s were employed as input for the functional analysis.

#### 5.2.4. Identification of Pathway-Based Bacterial Key Genes (bKGs) from DABCs

The content of functional genes in the gut microbiota was predicted using Phylogenetic Investigation of Communities by Reconstruction of Unobserved States 2 (PICRUSt2) software based on the identified features (differentially abundant bacterial compositions) [112]. PICRUSt2 predictions were based on the enzyme classification numbers and the Kyoto Encyclopedia of Genes and Genomes (KEGG) orthologs (KO) (v77.1). This functional analysis was utilized to investigate all of the samples’ metabolic pathways and genes. After that, we used Statistical Analysis of Metagenomic Profiles (STAMP) software [113] to identify differentially abundant metabolic pathways between SARS-CoV-2 and control patients and then collected all genes from these identified pathways. To identify differential metabolic pathways between the two groups, we utilized Welch’s *t* statistic to produce q-values for the two-tailed test, and Welch’s inverted confidence interval method was applied to calculate the confidence interval. The Benjamini–Hochberg false discovery rate method was then applied to compute the adjusted *p*-value (*p* < 0.05). Then, microbial protein–protein interaction networking of those pathway genes was performed by using the STRING database [114], and the result was visualized by using the cytoscape software [115] to identify the top-ranked bacterial key genes (bKGs) that were used as drug targets for further molecular docking analysis (see Figure S1).

#### 5.2.5. Bacterial Key Genes Guided Drug Repurposing by Molecular Docking

Molecular docking analysis was performed to repurpose potential drug molecules by using AutoDock Vina [116] for the treatment against bacterial co-/super-/secondary infections with SARS-CoV-2 infections. Initially, all receptor protein structures mediated by bKGs were downloaded from the Protein Data Bank (PDB), and heteroatoms, water molecules, and attached ligands were removed using BIOVIA Discovery Studio [117] and PyMOL [118]. Subsequently, protein energies were minimized with SwissPdb Viewer [119] and finally converted to an acceptable PDBQT format by fixing the grid box center in AutoDock tools. The ligand structures were energy-minimized using the MMFF94 force field in the Avogadro software [120] with specific parameters (total steps: 200, update steps: 1, energy difference: 0.1) [121]. Minimized ligands were processed and then converted to the PDBQT format using AutoDock tools, and protein–ligand complexes had their ionizable group pKa values estimated with PROPKA at physiological pH 7.0 [122]. Docking analysis was performed using AutoDock Vina (version 1.1.2) with an exhaustiveness parameter set to 10. We visualized the top-ranked drug–target complexes by arranging ligands and targets based on descending average binding affinity scores (kcal/mol) using BIOVIA Discovery Studio (version 3.0) and PyMOL software (version 2.3) to verify non-bond interactions. Furthermore, we conducted pharmacokinetic and toxicological assessments of the top-ranked drug molecules using their canonical SMILES as input on three online web servers: admetSAR [123], SwissADME [124], and pKCSM [125].

To evaluate the overall stability of the top three complexes, molecular dynamic simulations were conducted using YASARA software [126] and the AMBER14 force field [127], neutralized at 298 K, pH 7.4, and 0.9 percent NaCl. Energy minimization and annealing methods were applied before simulating for 100 ns, with trajectory snapshots saved every 100 ps. MM-Poisson–Boltzmann Surface Area (MM-PBSA) binding free energy for the complexes was calculated using captured trajectory snapshots [128].

Binding Energy = E_potRecept_ + E_solvRecept_ + E_potLigand_ + E_solvLigand_ − E_potComplex_ − E_solvComplex_

**Figure 9:**
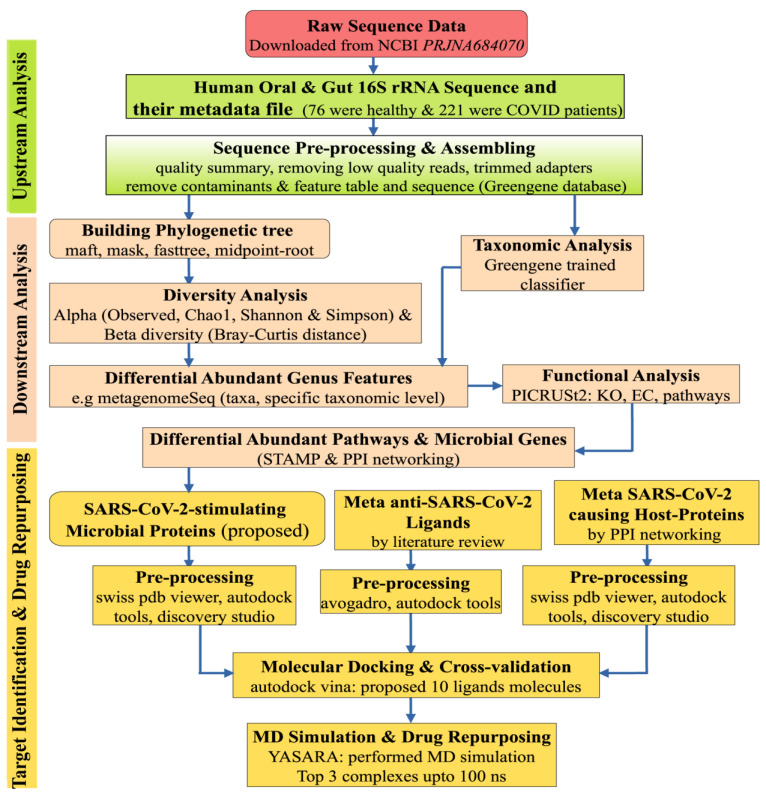
The workflow of the entire study involves different stages: upstream analysis is represented by green color, downstream analysis by burlywood color, and molecular docking and molecular dynamic simulation study by gold color.

In this study, the MM-PBSA binding free energy was calculated for the top-ranked 3 complexes using the YASARA macro, where positive energy signifies better binding [129]. Simulation snapshots were used to calculate the root-mean-square deviation (RMSD), the radius of gyration, and the surface-accessible surface area (SASA) [130,131,132,133,134]. The entire workflow of this study is displayed in Figure 9.

## 6. Conclusions

This study attempted to explore co-/super-/secondary infection-causing bacterial pathogens and their inhibitors for SARS-CoV-2 infection by using integrated statistical and bioinformatics approaches. At first, we analyzed the 16S rRNA bacterial sequence profiles of 297 individuals, of which 221 were COVID-19 patients and 76 were healthy, using Qiime2. It produced an OTU table of the count data between bacteria and patient samples. The diversity analysis based on the OTU table showed that SARS-CoV-2-infected samples are significantly separated from the control samples by the changes in the bacterial community. Several differentially abundant genus-taxa (*Bacteroides*, *Parabacteroides*, *Prevotella Clostridium*, *Atopobium,* and *Peptostreptococcus)* between SARS-CoV-2 infection and control samples were identified by using the OUT-table with the taxon-data table in the metagenomeSeq r-package. Then, we detected 23 significantly differentially abundant metabolic pathways based on the differentially abundant bacterial genus by using STAMP software. Among them, ‘incomplete reductive TCA’ and ‘pyruvate fermentation to acetate and lactate II’ pathways were significantly upregulated: palmitate biosynthesis II, palmitoleate biosynthesis I, and oleate biosynthesis IV pathways, were significantly downregulated. We obtained 183 bacterial genes from the identified differentially abundant pathways. Then, we selected the top-ranked 10 bKGs (*accB*, *ftsB*, *glyQ*, *hldD*, *lpxC*, *lptD*, *mlaA*, *ppsA*, *ppc*, and *tamB*) by the protein–protein interaction (PPI) network analysis of those 183 pathway genes. Then, we detected the bKGs-guided top-ranked 10 anti-SARS-CoV-2 drug molecules by molecular docking. Similarly, the SARS-CoV-2 infection-causing top-ranked nine published key gene (*ACE2*, *RdRp*, *3CLpro*, *S*, *TMPRSS2*, *PLpro*, *IL6*, *TNF*, and *N*)-guided the top-ranked 10 anti-SARS-CoV-2 drug molecules were also identified. Then, we considered the top-ranked eight common molecules (Bemcentinib, Ledipasvir, Velpatasvir, Tirilazad, Acetyldigitoxin, Entreatinib, Digitoxin, and Elbasvir) out of ten as the candidate anti-SARS-CoV-2 drug molecules. Finally, the binding stability of the top-ranked three drug–target complexes was investigated by molecular dynamic (MD) simulation studies and found to be stable. Our in silico pipeline might be a useful guideline to explore potential targets and ligands for taking a proper treatment plan against microbial co-/secondary infections with other diseases as well.

## Figures and Tables

**Table 1:** The top 20 differentially abundant gut microbiomes between COVID-19 and healthy patients were taken based on the adjusted *p*-value and log2FC value. The MetagenomeSeq R package was used to perform the analysis based on the zero-inflated Gaussian method. Among them, nine bacterial genera were upregulated, and the remaining eleven genera were downregulated.

Phylum	Family	Genus	Species	log2FC	Adj. *p*-Value
Proteobacteria	Neisseriaceae	*Neisseria*	*oralis*	−4.42	0.000
Firmicutes	Streptococcaceae	*Streptococcus*	*infantis*	−4.32	0.000
Firmicutes	Gemellaceae	*Unclassified*	*Unclassified*	−4.29	0.000
Firmicutes	Streptococcaceae	*Streptococcus*	*Unclassified*	−4.15	0.000
Fusobacteria	Leptotrichiaceae	*Leptotrichia*	*Unclassified*	−4	0.000
Proteobacteria	Oxalobacteraceae	*Cupriavidus*	*Unclassified*	−3.8	0.000
Firmicutes	Veillonellaceae	*Veillonella*	*parvula*	−3.69	0.000
Proteobacteria	Pasteurellaceae	*Unclassified*	*Unclassified*	−3.63	0.000
Firmicutes	Unclassified	*Unclassified*	*Unclassified*	−3.52	0.000
Proteobacteria	Burkholderiaceae	*Lautropia*	*Unclassified*	−3.49	0.000
Proteobacteria	Pasteurellaceae	*Haemophilus*	*influenzae*	−3.01	0.000
Firmicutes	Unclassified	*Unclassified*	*Unclassified*	3.08	0.000
Bacteroidetes	Porphyromonadaceae	*Parabacteroides*	*gordonii*	3.18	0.000
Actinobacteria	Actinomycetaceae	*Actinomyces*	*hyovaginalis*	3.24	0.000
Proteobacteria	Campylobacteraceae	*Campylobacter*	*fetus*	3.26	0.000
Firmicutes	Lachnospiraceae	*Clostridium*	*difficile*	3.49	0.000
Bacteroidetes	Prevotellaceae	*Prevotella*	*melaninogenica*	3.62	0.000
Actinobacteria	Coriobacteriaceae	*Atopobium*	*Rimae*	3.87	0.000
Firmicutes	Peptostreptococcaceae	*Peptostreptococcus*	*Anaerobius*	3.95	0.000
Bacteroidetes	Bacteroidaceae	*Bacteroides*	*Acidifaciens*	5.1	0.000

## Data Availability

The raw 16S rRNA sequence profile data set analyzed in this study is publicly available. It can be freely downloaded from the online NCBI database with bio-project number PRJNA684070. https://www.ncbi.nlm.nih.gov/bioproject/PRJNA684070 (access on 25 May 2023).

## Supplementary Materials

The following supporting information can be downloaded at: https://www.mdpi.com/article/10.3390/ph17040432/s1. Table S1: Comparison of different alpha diversity indices of bacterial communities with a significant difference between COVID-19 patients (*n* = 221) and healthy subjects (*n* = 76) based on Wilcoxon-Mann–Whitney test *p* values; Table S2: Comparative relative abundances of the oral and gut microbiota at the phylum and genus levels in the COVID-19 and healthy groups. Firmicutes, Bacteroidetes, Proteobacteria, and Fusobacteria were the most abundant phyla between the two groups, whereas Bacteroides, Prevotella, Actinomyces, Rothia, and Fusobacterium were the most dominant genera in the microbiome of the sample; Table S3: Pharmacological assessment of the top three potential ligand molecules derived from the admetSAR, SwissADME, and pKCSM web servers; Table S4: Metadata of SARS-CoV-2 infection obtained by reviewing published articles were used in this study; Table S5: Binding affinity score of two FDA-approved drugs corresponding to our proposed and published reviewed targets; Table S6: Non-bond interactions between top-ordered three receptors and ligand compounds based on their binding affinity; Figure S1: A workflow for exploring potential functional genes and their metabolic pathways from differentially abundant bacterial genera between SARS-CoV-2 infection and control samples. All the references in the supplementary materials have been mentioned in this paper.
