# Supplementary material for: Discovery of Bacterial Key Genes from 16S rRNA-Seq Profiles That Are Associated with the Complications of SARS-CoV-2 Infections and Provide Therapeutic Indications
Source: Pharmaceuticals (Basel). 2024 Mar 28;17(4):432. doi: 10.3390/ph17040432 (PMC11053588; doi:10.3390/ph17040432)
Supplement: Supplementary file 1 [file pharmaceuticals-17-00432-s001.zip › pharmaceuticals-2911417-supplementary.pdf]

Topic: Discovery of bacterial key genes from 16s rRNA-seq profiles that are associated with the complications of SARS-CoV-2 infections and provide therapeutic indications

(Supplementary File)

Tables/Figures	Contents	Pages
**Table S1**	Comparison of different alpha diversity indices of microbial communities with a significant difference between COVID-19 patients (n=221) and healthy subjects (n=76) based on Wilcoxon-Mann-Whitney test p values.	2
**Table S2**	Comparative relative abundances of the oral and gut microbiota's at the phylum, and genus level in COVID-19 and healthy group. Firmicutes, Bacteroidetes, Proteobacteria and Fusobacteria were the most abundant phyla between two group whereas Bacteroides, Prevotella, Actinomyces, Rothia and Fusobacterium were the most dominated genera's in the microbiome of the sample.	2-3
**Table S3**	Pharmacological assesment of the top three potential liggand molecules derived from the admetSAR , SwissADME, and pKCSM web-servers.	3-4
**Table S4**	Metadata of SARS-CoV-2 infections obtained by reviewing published articles were used in this study	4-15
**Table S5**	Binding affinity score of two FDA-Approved drug corresponding to our proposed and published reviewed targets.	16
**Table S6**	Non-bond interactions between top-ordered three receptors and ligand compounds based on their binding affinity.	16-18
**Figure S1**	A workflow of exploring potential functional genes and their metabolic pathways from differentially abundant bacterial genus between SARS-CoV-2 infection and control samples.	18
**Reference**		19-25

Table S1: Comparison of different alpha diversity indices of microbial communities with a significant difference between COVID-19 patients (n=221) and healthy subjects (n=76) based on Wilcoxon-Mann-Whitney test p values.

Alpha Diversity	Disease Status	Wilcoxon-Mann-Whitney test			Cliff's Delta	
		Median (IQR)	P-value (FDR)		Mean (SD)	Effect size
Observed	Patients	937.5 (717.25, 1199.75)	2.2e-16		3105.36 (825.81)	0.983 (large)
	Healthy	2941.5 (2502, 3874)			1003.21 (431.24)	
Chao1	Patients	1729.328 (1303.84, 2200.93)	2.2e-16		5128.32 (1222.61)	0.987 (large)
	Healthy	4852.912 (4250.33, 6318.36)			1807.16 (688.06)	
Shannon	Patients	4.145 (3.605, 4.556)	3.442e-15		4.84 (0.612)	0.606 (large)
	Healthy	4.875 (4.455, 5.263)			4.00 (0.795)	
Simpson	Patients	0.953 (0.919, 0.971)	3.453e-05		0.962 (0.023)	0.319 (small)
	Healthy	0.969 (0.954, 0.977)			0.942 (0.038)	

Table S2: Comparative relative abundances of the oral and gut microbiota's at the phylum, and genus level in COVID-19 and healthy group. Firmicutes, Bacteroidetes, Proteobacteria and Fusobacteria were the most abundant phyla between two group whereas Bacteroides, Prevotella, Actinomyces, Rothia and Fusobacterium were the most dominated genera's in the microbiome of the sample.

Taxonomic Rank	COVID-19 Patients			Healthy		Chi-square	*P-value*
	Median	***IQR***		***Median***	***IQR***		
**Phylum**							
Bacteroidetes	466	313.25-602.5		255	117-418	44.223	**2.93e-11**
Thermi	0.00	0.00-0.00		0.00	0.00-0.00	10.167	**0.001**
SR1	0.00	0.00-1.00		0.00	0.00-0.00	1.7739	0.182
TM7	0.00	0.00-0.00		1.0	0.0-10.0	49.122	**2.405e-**

							**12**
Actinobacteria	51.5	11-150.25		31.0	7.75-76.5	4.272	**0.038**
Proteobacteria	13	4.0-31.75		123	43.25-217	96.193	**2.2e-16**
Verrucomicrobia	0.00	0.00-0.00		0.00	0.00-0.00	16.752	**4.26e-05**
Cyanobacteria	0.00	0.0-1.0		0.00	0.0-2.0	11.43	**0.0007**
Firmicutes	61	36.25-144.75		286	217-377.5	72.77	**2.2e-16**
Fusobacteria	63	5.25 -132.5		43	16-85	4.913	**0.026**
**Genus**							
Porphyromonas	0.50	0.00-13.75		1.5	0.00-11.25	0.173	0.677
Bacteroides	47.50	13.0-224.75		24	6.75 -211.5	1.018	0.312
Prevotella	**165**	7.25 -375.5		64.5	22-177.25	12.89	**0.000**
Campylobacter	10.0	2.0-28.0		0.00	0.00-22.25	39.683	**2.987e-10**
Streptomyces	0.00	0.00-0.00		0.00	0.00-0.00	0.699	0.402
Bifidobacterium	0.00	0.00-1.00		0.00	0.00-2.0	1.781	0.181
Actinomyces	8.50	4.00-26.5		2.50	0.00-16.25	8.753	**0.003**
Rothia	4.00	0.00-23.75		2.00	0.00-8.0	6.856	**0.008**
Corynebacterium	**1.00**	0.0-10.0		2.00	0.00-22.25	3.375	**0.066**
Faecalibacterium	0.00	0.00-3.0		1.00	0.00-38.5	5.729	**0.016**
Fusobacterium	5.00	0.0-30.0		8.50	0.00-46.25	0.574	0.448
Clostridium	0.00	0.00-0.00		0.00	0.00-0.00	6.331	**0.011**

Values are median (IQR) of percentage relative abundance means.
***Kruskal-Wallis H test. P < 0.05 in bold.**

Table S3: Pharmacological assesment of the top three potential liggand molecules derived from the admetSAR , SwissADME, and pKCSM web-servers.

Parameter	Bemcentinib	Ledipasvir	Velpatasvir
Molecular weight (g/mol)	506.64	889.02	883.02
H-Bond Acceptor	8	8	9
H-Bond Donor	2	4	4
CNS	-2.04	-2.995	-3.597
CYP2D6 substrate	No	No	No
CYP3A4 substrate	Yes	Yes	Yes

Parameter	Bemcentinib	Ledipasvir	Velpatasvir
CYP1A2 inhibitor	No	Yes	No
CYP2C19 inhibitor	Yes	No	No
CYP2C9 inhibitor	Yes	No	No
CYP2D6 inhibitor	No	No	No
CYP3A4 inhibitor	Yes	No	Yes
Carcinogenicity	Non-carcinogenic	Non-carcinogenic	Non-carcinogenic
Hepatotoxicity	No	No	No
P-glycoprotein inhibitor	No	No	No
Acute Oral Toxicity	No	No	No

Table S4: Metadata of SARS-CoV-2 infections obtained by reviewing published articles were used in this study

Publication	Target	Candidate drugs
Rodrigues, L., et al. 2022[1]	S, E, N, M, NSP1,NSP2, NSP3, NSP4, NSP5, NSP6, NSP7, NSP8,NSP9, NSP10,ACE2	Chloroquine, Hydroxychloroquine, Alpha-1-proteinase inhibitor, Aluminium, Aluminium acetate, Aluminium phosphate, Aprotinin, Cyproterone acetate, Filgrastim, Mifepristone, Pegfilgrastim
Siminea, N. et al. 2022[2]	ACTB, AKT1, ATM, ATP6AP1, CSNK2A1, CDK2, EGF, MAPK14, MTOR and TP53	Bosutinib, brigatinib, dasatinib, imatinib, nilotinib, ponatinib, regorafenib, Dabrafenib, encorafenib, regorafenib, ripretinib, sorafenib, vemurafenib, Bosutinib, Afatinib, brigatinib, dacomitinib, erlotinib, gefitinib, icotinib, lapatinib, neratinib, olmutinib, osimertinib, vandetanib, zanubrutinib, Brigatinib, Entrectinib, fedratinib, ruxolitinib, zanubrutinib, Dasatinib, Everolimus, ridaforolimus, temsirolimus, Midostaurin
Khataniar, A., et al. 2022[3]	S, E, N, M, NSP1,NSP2, NSP3, NSP4, NSP5, NSP6, NSP7, NSP8,	Ascorbyl palmitate, cinametic acid, lauric acid, guaifenesin, nabumetone, nafcillin, octacosanol, palmidrol, and salmeterol, Dipyridamole, candesartan cilexetil, candesartan, oxytetracycline, valganciclovir hydrochloride, roxatidine acetate, omeprazole, sulfacetamide, cimetidine, disulfiram, atazanavir, hydroxychloroquine, chloroquine, indinavir montelukast sodium,

Publication	Target	Candidate drugs
	NSP9, NSP10, NSP11, NSP12, NSP13, NSP14, NSP15, NSP16, ACE2, RdRp	maribavir, Colchicine, remdesivir, bafilomycin A1, temozolomide, colchicine derivatives, Apamycin, camostat, nafamostat, saracatinib, trametinib, cefuroxime, ceftriaxone, cefotaxime, molnupiravir, grazoprevir, ganciclovir, atazanavir, daclatasvir, acyclovir, etravirine, entecavir, efavirenz, asunaprevir, abacavir dolutegravir, lomibuvir, penciclovir, trifluridine, danoprevir, ritonavir, saquinavir, raltegravir, lamivudine, Pemirolast, isoniazid pyruvate, nitrofurantoin, and eriodictyol
Islam, T., eta la. 2022[4]	S, ACE2, 3CLpro, RdRp, Mpro, Plpro,	Remdesivir, Molnupiravir, Paxlovid, Baricitinib
Wen, W. et al. 2022[5]	RdRp	molnupiravir, fluvoxamine and Paxlovid
McKee, D.L., 2022[6]	ACE2, 3Clpro, RdRp,TMPRSS2	Camostat mesilate, Nafamostat mesilate, Chloroquine phosphate, Hydroxychloroquine, Remdesivir, Lopinavir/ritonavir, Umifenovir, Favipiravir
Gysi, D.M. et al. 2021[7]	SIGMAR1, NSP6, CYP3A4, ABCB1, CYP2D6, CYP2C9, HTR2A, ALB	Ritonavir, Isoniazid, Troleandomycin, Cilostazol, Chloroquine, Rifabutin, Flutamide, Dexamethasone, Rifaximin, Azelastine, Crizotinib, Urea, Methylprednisolone, Dimethyl sulfoxide, Folic acid, Celecoxib, Betamethasone, Prednisolone, Mifepristone, Budesonide, Prednisone, Oxiconazole, Megestrol acetate, Idelalisib, Econazole, Rabeprazole, Quinine, Ticlopidine, Hydrocortisone, Lansoprazole, Methotrexate, Digoxin, Etoposide, Letrozole, Clobetasol, Quercetin, Tioguanine, Medroxyprogesterone acetate, Triamcinolone, Progesterone, Phenylbutyric acid, Colchicine, Teriflunomide, Dimethyl fumarate, Leflunomide, Bortezomid, Citalopram, Cholrambucil, Omeprazole, Ribavirin, Teniposide, Methimazole,

Publication	Target	Candidate drugs
		Cyclophosphamide, Acetic acid, Hydroxychloroquine, Ifosfamide, Aminoglutethimide, Fluconazole, Nelfinavir, Trabectedin, Bicalutamide, Theophylline, Enzalutamide, Gefitinib, Mebendazole, Adenosine, Mesalazine, Nevirapine, Belinostat, Mitomycine, Malathion, Ixekizumab, Vindesine, Secukinumab, Rifapentine, Bilastine, Nirmatrelvir , Clotrimazole, Erlotinib, Panobinostat, Warfarin, Busulfan, Goserelin, Hydroxyurea, Temsirolimus, Abiraterone, Miconazole, Ketorolac, Exemestane, Oxymetholone, Pentamidine, Diclofenac, Aminophylline, Loratadine, Fexofenadine, Terbinafine, Verapamil, Clopidogrel, Rivaroxaban
Chakraborty, C., 2021[8]	3CLpro, PLPro, RdRp, Mpro, hACE2	Chloroquine phosphate, Baricitinib, hydroxychloroquine sulfate, Azithromycin Lopinavir-Ritonavir,Tocilizumab, Favipiravir, Remdesivir, Baricitinib, Ivermectin,Dexamethasone,Baricitinib, Mavrilimumab, Hydroxychloroquine, Remdesivir, Dexamethasone, Tocilizumab, Mavrilimumab
Fangzhou Liu_2021[9]	AKT1, TP53, TNF, IL6, BCL2L1 and ATM	Matrine
Gurudeeban Selvaraj et al. 2021[10]	MYC, HDAC9, NCOA3, CEBPB, VEGFA, BCL3, SMAD3, SMURF1, KLHL12, CBL, ERBB4, and CRKL	Wortmannin
Ahmed, S. 2020[11]	ORF1ab , N, RdRP, E	IFN-α, Lopinavir/ritonavir, Ribavirin,Chloroquine phosphate, Arbidol
Muarya, V.K. et al. 2020[12]	RdRp, 3CLpro, Spike glycoprotein	Favipiravir, Ribavirin,Penciclovir, Remdesivir,Lopinavir, Ritonavir, Darunavir and cobicistat, ASC09F, Nafamostat, Griffithsin,Arbidol Oseltamivir,Chloroquine,Nitazoxanide
Huang, F. et al., 2020[13]	spike protein, ACE2,	Remdesivir/GS-5734, Chloroquine and

Publication	Target	Candidate drugs
	TMPRSS2, 3CLpro, RdRp and PLpro	Hydroxychloroquine, Lopinavir/Ritonavir(Kaletra) Favipiravir, EIDD-2801, Baricitinib, Methylprednisolone, Heparin, Zinc, Arbidol/Umifenovir, Darunavir, Oseltamivir, Emtricitabine<br>Tenofovir, Baloxavir marboxil, Danoprevir, Dipyridamole, Fingolimod Losartan, Azithromycin, Ribavirin, Triazavirin, Tranilast, Ebastine
EI-Din Abuo-Rahman, G.A., et al. 2020[14]	ACE2, TMPRSS2, ORF1a, ORF1b, Plpro, 3CLpro, RdRp	Favipiravir, Ribavirin,Penciclovir, Remdesivir (GS-5734),Galidesivir (BCX4430), 6′-Fluorinated-aristeromycin analogues Acyclovir fleximer analogues
Nitulescu, G.M. et al. 2020[15]	RdRp, S, PKR, N, M, TLR3, ACE2	Tocilizumab, Sarilumab, Siltuximab, Clazakizumab, Anakinra, Canakinumab XPro1595, Adalimumab, IFN-α 2a, Pegylated IFN-λ, IFN-β
Taz et al. 2020[16]	SAA2, MMP9, SAA1, S100A8, ICAM1, PI3, SOD2, C8orf4, SERPINA3, S100A12, S100A9, VEGFA, AKT1, MMP9, ICAM1, CD44	MIGLITOL CTD 00002031, CHEMBL55802 CTD 00003118, Hesperidin CTD 00006087, Cytochalasin D CTD 00007076, Prolinedithiocarbamate CTD 00002658, Parthenolide CTD 00000087, FEXOFENADINE HYDROCHLORIDE CTD 00003191, Hydroxytyrosol CTD 00000267, Antimycin A CTD 00005427, Anacardic acid C15:3 CTD 00003117,
Moni et al. 2020[17]	MX1, IRF7, BST2, BCL2A1, CSF2, EPSTI1, MMP13, CXCL6, OAS2, CXCL1, CXCL2, CXCL3, IFI6, IFI27 and TNF	Cytochalasin D, 1′-acetoxychavicol acetate, Atorvastatin, Proline dithiocarbamate, Dicumarol, Oleanolic acid.
Zhen-Zhen[18]	ACE2, TMPRSS2, IL-1α, IL-8, IL-6, and CCL-2, 3CLpro3	berberine/NIT-X
Yi-Wei Zhu et al. 2020 [19]	RELA, TNF, IL6, IL1B, MAPK14, TP53, CXCL8, MAPK3, MAPK1, IL4, MAPK8, CASP8, STAT1	Quercetin, Kaempferol, bsitosterol, Isorhamnetin, Naringenin, Luteolin, (þ)-catechin, Delphinidin, aloe-Emodin, Baicalein and Irisolidone
Suresh Kumar _et	VEGFA, TNF, IL-6,	Chloroquine, lenalidomide, Penicillin,

Publication	Target	Candidate drugs
al. 2020 [20]	CXCL8, IL10, CCL2, IL1B, TLR4, ICAM1, MMP9	Pentoxifylline, Thalidome, Sorafenib, Paclitaxel, Rapamycin, Cortisol, Statins
Zulkar Nain et al. 2020[21]	APC, KPNA2, CTNNB1, CCND1, NFKB1, FOS, HDAC2, PTPRK, LHB, FUS, CTBP1, THRA, CDX2 and BUB1	Antibiotic K-252A,Cabozantinib, Amuvatinib, Crizotinib, SGX-523, 888719-03-7, CHEMBL527066, CHEMBL503090, SCHEMBL15322421, CHEMBL462712, rac-crizotinib, CHEMBL561660, Crizotinib, Cabozantinib.
Li Zhonglin et al, 2020[22]	DYNLL1, POLR2F, RPL13A, FBXO11, CSNK1E	Podophyllotoxin, Amantadine, Thioperamide, Monensin, Vancomycin, Etiocholanolone, Acyclovir, Isoflupredone, Heptaminol,Chenodeoxycholic acid, Podophyllotoxin, Atractyloside, Adiphenine, Monensin, Lisuride
Islam et al. 2020[23]	BIRC3, ICAM1, IRAK2, MAP3K8, S100A8, SOCS3, STAT5A, TNF, TNFAIP3, TNIP1, FOXC1, GATA2, YY1, FOXL1, NFKB1	SYK-inhibitor, Radicicol, Dabrafenib, AT-7519, Dasatinib, Lovastatin, Thiostrepton, Linifanib, JNK–IN–5A, Withaferin-A.
Ge C et al 2020[24]	MMP13, NLRP3, TRIM21, GBP1, ADORA2A, PTAFR, TNF, MLNR, IL1B, NFKBIA, ADRB2, and IL6	Astragaloside IV
Aishwarya et al. 2020[25]	MDH1, SGCE, PFKFB3, PGM5, ISLR and ANK2	F-1566-0341, Digoxin, Proscillaridin, Linifanib
Tao et. al., 2020[26]	MAPK3, MAPK8, TP53, CASP3, IL6, TNF, MAPK1, CCL2, PTGS2	Quercetin, Kaempferol, Beta-sitosterol, Stigmasterol, Isorhamnetin, Baicalein, Naringenin, Formononetin
Beck et al. [27]	RdRp, 3CLpro	5-nonyloxytryptamine, Abacavir sulfate, Abacavir, Acetylcholine Chloride, Acyclovir, Adefovir Dipivoxil, Amprenavir (agenerase), Apixaban, Asunaprevir (BMS-650032), Atazanavir sulfate (BMS-232632-05), Atazanavir, Atropine, avermectin, Batimastat, Boceprevir, bosutinib, Cidofovir, Cyclosporine, dacinostat, Daclatasvir (BMS-790052), danoprevir, Daptomycin,

Publication	Target	Candidate drugs
		Darunavir, demecarium, Difloxacin HCl, dinoprostone, efavirenz, elvitegravir, Entecavir Hydrate, entecavir, eprosartan, Etomidate, everolimus, Famciclovir, foxy-5, Ganciclovir, indinavir, ivermectin, Leuprolide Acetate, lisuride, lopinavir, Methscopolamine, mupirocin, naltrindole, Nelfinavir Mesylate, nelfinavir, nevirapine, Octreotide acetate, oligomycin-a, Oseltamivir acid, Oseltamivir phosphate, Oseltamivir, Otilonium Bromide, Penciclovir, Peramivir Trihydrate, Peramivir, Pimecrolimus, prostaglandin, Radotinib(IY-5511), raltegravir, Rapamycin (Sirolimus), Remdesivir, ribavirin, Rifabutin, Rilpivirine, Ritonavir, Rupatadine Fumarate, Saquinavir mesylate, saquinavir, saracatinib, scopolamine, Sildenafil Citrate, sirolimus, somatostatin, Tacrolimus (FK506), Telaprevir (VX-950), temsirolimus, Tenofovir Disoproxil Fumarate, tenofovir, thiostrepton, Tigecycline, Tiotropium Bromide, torin-2, trichostatin-a, Valaciclovir HCl, valaciclovir, Valganciclovir HCl, Zanamivir, zolmitriptan, PHA-665752
Kartikay Prasad et al. 2020[28]	IFIT1, IFITM1, IRF7, ISG15, MX1, and OAS2, TLR3,	Mitomycin-C, Imiquimod, Polyinosinic:polycytidylic acid (poly I:C),S-carbamidomethylcysteine(Cysteine-S-acetamide), Vanadium oxide and MgATP.
Han et. al, 2020 [29]	IL6, ACE2, TNF, IL10, MAPK8, MAPK3, CXCL8, CASP3, PTGS2, TP53, and MAPK1	Quercetin, Luteolin
S. A. Khan et al., 2021 [30]	3CLpro	Remdesivir, Saquinavir, Darunavir, flavone, coumarin derivatives
Alves et al., 2021 [31]	M^{pro}	Sufugolix, Cenicriviroc, Proglumetacin
Umesh et al., 2021[32]	M^{pro}	Alpha-ketoamide, Carnoso, Rosmanol, Arjunglucoside-I
Liu et al., 2020[33]	3CLpro	Itacitinib, Oberadilol, Telcagepant,

Publication	Target	Candidate drugs
		Vidupiprant, Pilaralisib, Poziotinib, Fostamatinib, CL-275838, Ziprasidone, Leucal/Folinic Acid, ITX5061
Jin et al., 2020[34]	M^{pro}	Ebselen, Shikonin, Tideglusib, PX-12, TDZD-8, Carmofur
Feng et al., 2021[35]	$3CL^{Pro}$	antiviral drugs (lopinavir, tenofovir disoproxil, fosamprenavir and ganciclovir), antif lu drugs (peramivir and zanamivir) and an anti-HCV drug (sofosbuvir)
Günther et al., 2021[36]	M^{pro}	Adrafinil, AR-42, AT7519, Aurothioglucose, AZD6482, Bromebric acid, Calpeptin, Climbazole, Clonidine, Dexrazoxane, Fusidic acid, Glutathione monoisopropyl ester, Glycitein, BE-2254, Ifenprodil, Ipidacrine, Isofloxythepin, LSN2463359, Maleic acid, Neptazane, MUT056399, Necrostatin-1, PD 168568, Pelitinib, Polydatin, RS102895, SEN-1269, Suxamethonium, SUN-B 8155, Tegafur, Evofosfamide, Tofogliflozin, Tolperisone, Tretazicar, Teroxirone, UNC-2327, Zinc pyrithione
R. J. Khan et al., 2021[37]	3CLpro, 2'-O-MTase	Raltegravir, Paritaprevir, Bictegravir and Dolutegravir
Raj, 2021[38]	NSP-3, 5, 11, 14, 15	DB01977, BD07132, DB07535
Kuo et al., 2021[39]	$3CL^{pro}$, PL^{pro}	levothyroxine, manidipine-2HCl levothyroxine, loperamide, manidipine-2HCl, maprotiline, reserpine, proanthocyanidin
Anand et al., 2021[40]	NSP10, Nucleoprotein, NSP3, 3CLpro,	Darifenacin, Nebivolol, Bictegravir, Alvimopan, Irbesartan,
Cavasotto and Di Filippo, 2021[41]	M^{pro}, PL^{pro}, S-protein	ENMD-981693, Felypressin, Brilacidin, Samatasvir, Eribaxaban, Aplidin, Candesartan Cilexetil, Ritonavir, Tomivosertib, Rebamipide, Saquinavir, Flovagatran, Sovaprevir, Indinavir, Anatibant, Pilaralisib, Tiracizine, Zabofloxacin, Picotamide, Cilazapril, Indisulam, Darolutamide, Ziprasidone,

Publication	Target	Candidate drugs
		Propamidine, Pralatrexate, Carumonam, Aclerasteride, Granotapide
Gil et al., 2020[42]	RNA polymerase, 3CLpro, PLpro, S	remdesivir, favipiravir, ribavirin, oseltamivir, galidesivir, sofosbuvir, umifenovir, lopinavir/ritonavir, ivermectin, disulfiram, griffithsin
Guedes et al., 2021[43]	PLpro, Mpro, RdRp, N, S	Prazosin, Bazedoxifene, Menaquinone, Posaconazole, Ledipasvir, Imatinib, Atorvastatin, Ivermectin, Elbasvir, Velpatasvir, Pibrentasvir, Ombitasvir, Dactinomycin, Ribavirin, Daclatasvir, Ivermectin, Elbasvir, Fdaxomicin, Atorvastatin, Lomitapide
Liang et al., 2021[44]	RdRp, 3CLpro, PLpro, S	avapritinib, bictegravir, ziprasidone, capmatinib, pexidartinib
Rahman et al., 2021[45]	M^{pro}, RdRp, PL^{pro}, S	Rutin
Murugan et al., 2020[46]	3CLpro, Plpro, RdRp	Baloxavir marboxil, Phthalocyanine, Tadalafil, Lonafarnib, Nilotinib, Dihydroergotamine, R-428
Manikyam and Joshi, 2020[47]	3CLpro, Plpro,RdRp	paritaprevir, ritonavir, entecavir and chloroquine
Wu et al., 2020[48]	3CLpro, PLpro, RdRp	ribavirin, valganciclovir, thymidine, cefpiramide, sulfasalazine, phenethicillin, lymecycline, demeclocycline, doxycycline, oxytetracycline, tigecycline, montelukast, fenoterol
Abdel-Basset et al., 2020[49]	3CLpro, RdRp	Cilostazol, Baricitinib, Fluconazole, Itraconazole, Quercetin, Rabeprazole, Grazoprevir, Sirolimus, Ivermectin, Methylprednisolone, Abacavir, Rifaximin, Ritonavir, Metoprolol , Digoxine
Mishra et al., 2021[50]	S, hACE2, 3CLpro, CTSL, nucleocapsid protein, RdRp, NSP6	Ritonavir, Dolutegravir, Tenofovir, Tinofoviralafenamide, Boceprevir, Catechin, Zanamivir
Nelakuditi and Shrivastava, 2020[51]	Mpro, S, ACE2, RdRp	Vilazodone, Lopinavir, Ritonavir, Darunavir, Selinexor, Etoposide, Nintedanib, Methylprednisolone, Hydrocortisone, Tolcapone, Apixaban, Rivaroxaban, Dabigatran, Betrixaban, Amprenavir

Publication	Target	Candidate drugs
Mhatre et al., 2021[52]	3CLpro, S, PLpro, RdRp, ACE2	Favipiravir
Joshi et al., 2020[53]	SARS-CoV-2 M^{Pro}, RdRp and hACE-2	δ-viniferin, Myricitrin, Taiwanhomoflavone A, Lactucopicrin 15-oxalate, Nympholide-A, Saquinavir, Biorobin, Phyllaemblicin B, -(-)asperlicin, Cassameridin, Chrysanthemin, Scalarane, Baicalin, Hesperidin, Afzelin
Shi et al., 2021[54]	ACE2, Mpro, RdRp	Val-Ser-Gly-Ala-Gly-Arg-Tyr, Val-Met-Asp-Lys-Pro-Gln-Gly, Val-Ile-Glu-Lys-Tyr-Pro, Lys-Asp-Tyr-Arg-Leu, Asp-GluAsn-Ser-Lys-Phe, Asn-Asn-Asn-Pro-Phe-Lys-Phe
Panda et al., 2020[55]	SARS-CoV-2 Mpro, S, RBD, ACE2	Lopinavir, ritonavir, zanamivir, Remdesivir, PC786, JNJ
Jena et al., 2021[56]	ACE2, Mpro	Alkaloid 1e, Steroids 3a, Terpenoids 4b, Terpenoids 4n, Octacosanol 5b and Heptacosanol 5c
Duverger et al., 2021[57]	ACE-2	Azithromycin, hydroxychloroquine
Xiang et al., 2021[58]	ACE2	puerarin
de Oliveira et al., 2021[59]	ACE2	Theaflavin digallate, suramin sodium, 5-hydroxytrytophan, solamargine, beta-solamargine, taraxanthin, anthranil acid, evomonoside, dihydroergocristine mesylate, smilacin, withaphysalin, erysimosol, quinupristin, tigogenin, sarsasapogenin, Nilotinib, nilotinib hydrochloride monohydrate, deacetylo-leandrin, dexamethasone-21-sulfobenzoate, dauricinoline, tirilazad, swertifrancheside, digitoxin, Digitoxin, selamectin, acetyldigitoxin, gitaloxin, doramectin
Bardaweel et al., 2021[60]	DPP4, ACE2	sitagliptin
Kabir et al., 2021[61]	ACE2, TMPRSS2	10-gingerdione, campesterol, cilastazol, dapagliflozin, danoprevir, dithymoquinone, doxycycline, edoxaban, empaflozin, englitazone, erythromycin, ezetimibe,fluvastatin, fucosterol, glimepiride, gemigliptin, glyburide, methyl prednisolone,

Publication	Target	Candidate drugs
		mevastatin, pacritinib, pitavastatin, prasugrel, rosiglitazone, shikonin, tideglusib, ticagrelor and warfarin.
Bojkova et al., 2020[62]	ACE2, RdRp	Cycloheximide, 2-deoxy-d-glucose, ribavirin, NMS-873
Aftab et al., 2020[63]	RdRp, ASP760, ASP761,	CID123624208, CID11687749,
Elfiky, 2021[64]	RdRp	Sofosbuvir, Ribavirin, Galidesivir, Remdesivir, Favipiravir, Cefuroxime, Tenofovir, Hydroxychloroquine, Setrobuvir, YAK, and IDX-184
Pirzada et al., 2021[65]	RdRp	Remdesivir, Ledipasvir
Agrawal et al., 2021[66]	S, RdRp	Indinavir, Nelfinavir, Fosamprenavir, Rintatolimod, Loviride, Nevirapine, Nitazoxanide, Imiquimod, Inosine, Cobicistat, Vancomycin, Gliclazide, Azithromycin, Sulfamethoxazole, Meropenem, Tenofovir, Disoproxil, Trimethoprim, Ciprofloxacin, Gentamicin, Levofloxacin, Ivermectin B1a, Ivermectin B1b, Hydroxychloroquine, Beclabuvir, Galidesivir, Ribavirin, Favipiravir, Sofosbuvir, Tenofovir, Remdesivir
Ruan et al., 2021[67]	NSP12-NSP7-NSP8	Nilotinib, Saquinavir, Tipranavir, Lonafarnib, Tegobuvir, Olysio, Filibuvir, and Cepharanthine
Y. J. Sun et al., 2021[68]	TMPRSS2	avoralstat
Cho et al., 2021[69]	TMPRSS2	glycyrrhizin
Gao et al., 2021[70]	PLpro	GRL0617
Zhao et al., 2021[71]	PLpro	YM155, Cryptotanshinone, Tanshinone I, GRL0617
Weglarz-Tomczak et al., 2021[72]	PL^{pro}	Ebselen
Sinha et al., 2021[73]	NSP15, S	Saikosaponins U and V
Yang et al., 2021[74]	SARS-CoV-2 S protein	Clemastine, Amiodarone,

Publication	Target	Candidate drugs
		Trimeprazine, Bosutinib, Toremifene, Flupenthixol, Azelastine
Jeon et al., 2020[75]	N protein	niclosamide, ciclesonide
Kumar et al., 2021[76]	Noscapines protease	Noscapine
C. Liu et al., 2021[77]	NSP14	Saquinavir, Hypericin, Baicalein, Bromocriptine
El Hassab et al., 2021[78]	nsp16	AP-20, Sinefungin,
G. Li et al., 2021[79]	TNF	Etanercept, Baclofen, adalimumab, tocilizumab, rituximab, glucocorticoids
F. Sun et al., 2021[80]	AKT1, AKT2 and AKT3	Capivasertib
Dittmar et al., 2021[81]	Cyclophilin	Salinomycin, Y-320, AZD8055, Bemcentinib, Dacomitinib, WYE-125132, Ebastine, Dp44mT, Cyclosporine
Han et al., 2021[82]	NFKBIA, IKBKB, CYP450	Sulfasalazine
Y. Li et al., 2021[83]	SRC, HDAC, MEK	Atorvastatin, Ibuprofen, Ketoconazole
Sauvat et al., 2020[84]	SARS-CoV-2	cephararanthine, chloroquine, hydroxychloroquine chloroquine, chlorpromazine, emetine, mefloquine
Krafcikova et al., 2020[85]	2′-O-RNA methyltransferase (MTase), nsp10-nsp16, RdRp, RNA cap	Sinefungin
Francis Borgio et al., 2020[86]	SARS-CoV-2 helicase,	Vapreotide, atazanavir
Díaz, 2020[87]	orf8, M, Nsp7, orf1b	Rapamycin, FK-506, Bafilomycin A1 Entacapone, Indomethacin, Metformin, Remdesivir
Gordon et al., 2020[88]	SIGMAR1	L-cloperastine, clemastine
Auwul et al., 2021[89]	PLK1, AURKB, AURKA, CDK1, CDC20, KIF11, CCNB1, KIF2C, DTL, CDC6	amsacrine, BRD-K68548958, naproxol, palbociclib, teniposide
Belyaeva et al., 2021[90]	HEK293T, ACAT1, ADK,AGA	Afatinib, axitinib, bosutinib, dasatinib, sorafenib, formoterol, docetaxel
Lee et al., 2021[91]	SLC3A2, SLC2A3, FOLR2	fluorodeoxyglucose
Desvaux et al.,	ST2,	Dexamethasone, Canakinumab, Siltuximab,

Publication	Target	Candidate drugs
2021[92]	RAGE	Sarilumab, Tocilizumab, Infliximab, Adalimumab, Hydrocortisone, Prednisolone, Baricitinib, Ruxolitinib, progesterone, Secukinumab, Eculizumab, Anakinra, Romiplostim
O'Donovan et al., 2021[93]	MEK inhibitor	Trametinib, Withaferin A, Parthenolide, Lapatinib, Sorafenib, Auranofn, Selumetinib
Yee et al., 2021[94]	OATP2B1, OCT1, OCT2, OAT1,OAT3, MATE1, and MATE2	Azithromycin, Baricitinib, Camostat, Chloroquine, Colchicine, Darunavir, Favipiravir, Fingolimod, Hydroxychloroquine, Leflunomide, Lopinavir, Losartan, Oseltamivir, Piclidenoson, Prazosin, Remdesivir, Ribavirin, Ritonavir, Ruxolitinib, Sildenafil, ,Tetrandrine, Thalidomide, Tofacitinib, Triazavirin, Umifenovir
Zhou et al., 2020[95]	ORF1ab, ACE2,JUN, XPO1, NPM1, HNRNPA1	Sirolimus, dactinomycin, mercaptopurine, melatonin, toremifene, emodin
Alam et al., 2021[96]	hsa-miR-1307-3p, hsa-miR-1912-5p, hsa-miR-766-3p, hsa-miR-1910-5p, hsa-miR-1304-5p	Miravirsen
Alanazi, Farah, and Hor 2022 [97]	NSP1, PLpro, Mpro, NSP9, RdRp, NSP13, NSP15, ORF3a, S, E, M, ORF6, ORF7a, N	Rutin, NADH, Ginsenoside Rg1, protopanaxatriol
Jose et al. 2022[98]	S	Astragalin, 4-p-Coumaroylquinic Acid, 3-p-Coumaroylquinic Acid, Sinapoyl-D-Glucoside, 1-Sinapoyl-D- Glucose
**Nine genes that are common at least 6 articles** (the rest of them are common at most 5 articles)	**ACE2** (common in 20 articles), **RdRp** (common in 30 articles), **3CLpro** (common in 22 articles), **S** (common in 18 articles), **TMPRSS2** (common in 7 articles), **Plpro** (common in 18 articles), **IL6** (common in 6 articles), **TNF** (common in 9 articles), **N** (common in 6 articles)	

Table S5: Binding affinity score of two FDA-Approved drug corresponding to our proposed and published reviewed targets.

**Protein Name**	**Molnupiravir**	**Nirmatrelvir**
**Proposed Protein**		
hldD	-7.8	-7.1
mlaA	-6.8	**-7.9**
lptD	**-8.1**	-5.7
accB	-5.3	-5.7
ftsB	-5.4	-6.2
glyQ	-6.9	-5.4
lpxC	-6.3	-7.2
ppc	**-8.3**	-6.5
ppsA	-6.8	-6.6
tamB	-6.0	-6.6
**Published Protein (taken by reviewing published articles)**		
3CLpro	-6.5	-6.5
ACE2	-6.6	-7.3
IL6	-5.7	-6.2
N	-5.6	-6.4
PLpro	-6.6	-7.4
RdRp	-7.4	-7.9
S	-6.0	-6.0
TMPRSS2	-7.2	-6.9
TNF	-5.8	-6.2

Table S6. Non-bond interactions between top-ordered three receptors and ligand compounds based on their binding affinity.

**PubChem CID**	**Drug compounds**	**Receptors/Targets**	**Binding affinity (kcal/mol)**	**Interacting Amio Acid**	**Interaction Type**	**Distance in Å**
46215462	Bemcentinib	hldD	-11.8	ARG213<br>PHE200<br>HIS186<br>TYR292<br>VAL168	Pi-Donor Hydrogen<br>Pi-Sigma<br>Pi-Pi T-shaped<br>Pi-Pi T-shaped<br>Alkyl	3.926<br>3.720<br>5.217<br>5.177<br>5.147

**Table S6.** Non-bond interactions between top-ordered three receptors and ligand compounds based on their binding affinity.						
**PubChem CID**	**Drug compounds**	**Receptors/Targets**	**Binding affinity (kcal/mol)**	**Interacting Amio Acid**	**Interaction Type**	**Distance in Å**
				LYS198	Pi-Alkyl	5.334
67505836	Ledipasvir	mlaA	-11.7	THR152<br>TRP169<br>GLY138<br>PRO135<br>GLY136<br>TYR137<br>LEU180<br>LEU157<br>VAL197	Conventional Hydrogen<br>Conventional Hydrogen<br>Carbon Hydrogen<br>Carbon Hydrogen<br>Carbon Hydrogen<br>Pi-Pi Stacked<br>Alkyl<br>Alkyl<br>Pi-Alkyl	2.067<br>2.602<br>3.658<br>3.582<br>3.464<br>5.728<br>5.314<br>4.616<br>5.163
67683363	Velpatasvir	lptD	-11.4	LYS239<br>HIS265<br>THR160<br>PHE161<br>PHE192	Conventional Hydrogen<br>Conventional Hydrogen<br>Carbon Hydrogen<br>Pi-Pi T-shaped<br>Pi-Pi T-shaped	2.727<br>2.333<br>3.482<br>4.768<br>5.135
145996610	*Molnupiravir*	lptD	-8.3	LYS234<br>ASN345<br>LYS735<br>GLY753<br>ASN341<br>PHE343<br>GLU733<br>LYS234<br>ASP344<br>ALA751	Conventional Hydrogen<br>Conventional Hydrogen<br>Conventional Hydrogen<br>Conventional Hydrogen<br>Conventional Hydrogen<br>Conventional Hydrogen<br>Conventional Hydrogen<br>Pi-Cation<br>Pi-Anion<br>Pi-Alkyl	1.935<br>2.112<br>2.725<br>2.800<br>1.947<br>2.886<br>1.919<br>4.117<br>4.920<br>4.871
155903259	Nirmatrelvir	mlaA	-8.1	SER139<br>GLU145<br>ASP144<br>ASP148<br>HIS205	Conventional Hydrogen<br>Conventional Hydrogen<br>Halogen (Fluorine)<br>Halogen (Fluorine)	2.550<br>2.659<br>3.475<br>3.533<br>5.081

**Table S6.** Non-bond interactions between top-ordered three receptors and ligand compounds based on their binding affinity.						
**PubChem CID**	**Drug compounds**	**Receptors/Targets**	**Binding affinity (kcal/mol)**	**Interacting Amio Acid**	**Interaction Type**	**Distance in Å**
					Pi-Alkyl	

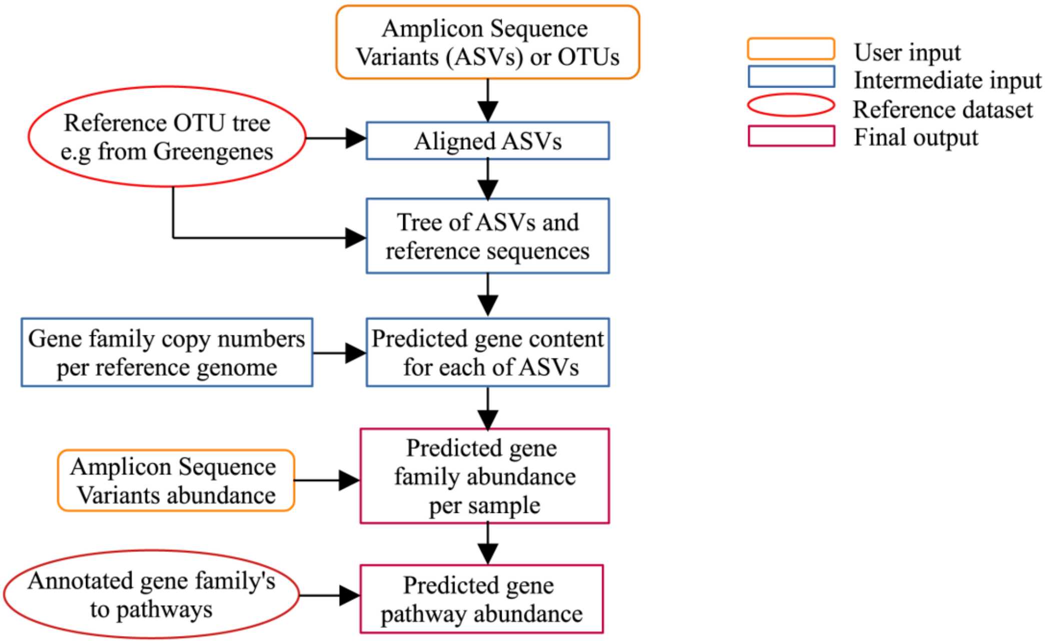

Figure S1. A workflow of exploring potential functional genes and their metabolic pathways from differentially abundant bacterial genus between SARS-CoV-2 infection and control samples.

Reference

1. Rodrigues, L.; Cunha, R.B.; Vassilevskaia, T.; Viveiros, M.; Cunha, C. Drug Repurposing for COVID-19: A Review and a Novel Strategy to Identify New Targets and Potential Drug Candidates. *Molecules* **2022**, *27*, doi:10.3390/molecules27092723.
2. Siminea, N.; Popescu, V.; Martin, J.A.S.; Florea, D.; Gavril, G.; Gheorghe, A.M.; Iţcuş, C.; Kanhaiya, K.; Pacioglu, O.; Popa, L.I.; et al. Network Analytics for Drug Repurposing in COVID-19. *Brief. Bioinform.* **2022**, *23*, doi:10.1093/bib/bbab490.
3. Khataniar, A.; Pathak, U.; Rajkhowa, S.; Jha, A.N. A Comprehensive Review of Drug Repurposing Strategies against Known Drug Targets of COVID-19. *Covid* **2022**, *2*, 148–167, doi:10.3390/covid2020011.
4. Islam, T.; Hasan, M.; Rahman, M.S.; Islam, M.R. Comparative Evaluation of Authorized Drugs for Treating Covid-19 Patients. *Heal. Sci. Reports* **2022**, *5*, doi:10.1002/hsr2.671.
5. Wen, W.; Chen, C.; Tang, J.; Wang, C.; Zhou, M.; Cheng, Y.; Zhou, X.; Wu, Q.; Zhang, X.; Feng, Z.; et al. Efficacy and Safety of Three New Oral Antiviral Treatment (Molnupiravir, Fluvoxamine and Paxlovid) for COVID-19 ： a Meta-Analysis. *Ann. Med.* **2022**, *54*, 516–523, doi:10.1080/07853890.2022.2034936.
6. McKee, D.L.; Sternberg, A.; Stange, U.; Laufer, S.; Naujokat, C. Candidate Drugs against SARS-CoV-2 and COVID-19. *Pharmacol. Res.* **2020**, *157*, doi:10.1016/j.phrs.2020.104859.
7. Gysi, D.M.; Do Valle, Í.; Zitnik, M.; Ameli, A.; Gan, X.; Varol, O.; Ghiassian, S.D.; Patten, J.J.; Davey, R.A.; Loscalzo, J.; et al. Network Medicine Framework for Identifying Drug-Repurposing Opportunities for COVID-19. *Proc. Natl. Acad. Sci. U. S. A.* **2021**, *118*, doi:10.1073/pnas.2025581118.
8. Chakraborty, C.; Sharma, A.R.; Bhattacharya, M.; Agoramoorthy, G.; Lee, S.S. The Drug Repurposing for COVID-19 Clinical Trials Provide Very Effective Therapeutic Combinations: Lessons Learned From Major Clinical Studies. *Front. Pharmacol.* **2021**, *12*, doi:10.3389/fphar.2021.704205.
9. Liu, F.; Li, Y.; Yang, Y.; Li, M.; Du, Y.; Zhang, Y.; Wang, J.; Shi, Y. Study on Mechanism of Matrine in Treatment of COVID-19 Combined with Liver Injury by Network Pharmacology and Molecular Docking Technology. *Drug Deliv.* **2021**, *28*, doi:10.1080/10717544.2021.1879313.
10. Selvaraj, G.; Kaliamurthi, S.; Peslherbe, G.H.; Wei, D.-Q. Identifying Potential Drug Targets and Candidate Drugs for COVID-19: Biological Networks and Structural Modeling Approaches. *F1000Research* **2021**, *10*, 127, doi:10.12688/f1000research.50850.1.
11. Ahmad, S. A Review of COVID-19 (Coronavirus Disease-2019) Diagnosis, Treatments and Prevention. *Eurasian J. Med. Oncol.* **2020**, doi:10.14744/ejmo.2020.90853.
12. Maurya, V.K.; Kumar, S.; Bhatt, M.L.B.; Saxena, S.K. Therapeutic Development and Drugs for the Treatment of COVID-19. **2020**, 109–126, doi:10.1007/978-981-15-4814-7_10.
13. Huang, F.; Li, Y.; Leung, E.L.H.; Liu, X.; Liu, K.; Wang, Q.; Lan, Y.; Li, X.; Yu, H.; Cui, L.; et al. A Review of Therapeutic Agents and Chinese Herbal Medicines against SARS-COV-2 (COVID-19). *Pharmacol. Res.* **2020**, *158*, doi:10.1016/j.phrs.2020.104929.
14. El-Din Abuo-Rahma, G.A.; Mohamed, M.F.A.; Ibrahim, T.S.; Shoman, M.E.; Samir, E.; Abd El-Baky, R.M. Potential Repurposed SARS-CoV-2 (COVID-19) Infection Drugs. *RSC Adv.* **2020**, *10*, 26895–26916, doi:10.1039/d0ra05821a.
15. Nitulescu, G.M.; Paunescu, H.; Moschos, S.A.; Petrakis, D.; Nitulescu, G.; Ion, G.N.D.; Spandidos, D.A.; Nikolouzakis, T.K.; Drakoulis, N.; Tsatsakis, A. Comprehensive Analysis of Drugs to Treat SARS-CoV-2 Infection: Mechanistic Insights into Current COVID-19 Therapies (Review). *Int. J. Mol. Med.* **2020**, *46*, 467–488, doi:10.3892/ijmm.2020.4608.
16. Taz, T.A.; Ahmed, K.; Paul, B.K.; Kawsar, M.; Aktar, N.; Mahmud, S.M.H.; Moni, M.A. Network-Based Identification Genetic Effect of SARS-CoV-2 Infections to Idiopathic Pulmonary Fibrosis

(IPF) Patients. *Brief. Bioinform.* **2020**, *00*, 1–13, doi:10.1093/bib/bbaa235.
17. Moni, M.A.; Quinn, J.M.W.; Sinmaz, N.; Summers, M.A. Gene Expression Profiling of SARS-CoV-2 Infections Reveal Distinct Primary Lung Cell and Systemic Immune Infection Responses That Identify Pathways Relevant in COVID-19 Disease. *Brief. Bioinform.* **2020**, *00*, 1–14, doi:10.1093/bib/bbaa376.
18. Wang, Z.Z.; Li, K.; Maskey, A.R.; Huang, W.; Toutov, A.A.; Yang, N.; Srivastava, K.; Geliebter, J.; Tiwari, R.; Miao, M.; et al. A Small Molecule Compound Berberine as an Orally Active Therapeutic Candidate against COVID-19 and SARS: A Computational and Mechanistic Study. *FASEB J.* **2021**, *35*, doi:10.1096/fj.202001792R.
19. Zhu, Y.W.; Yan, X.F.; Ye, T.J.; Hu, J.; Wang, X.L.; Qiu, F.J.; Liu, C.H.; Hu, X.D. Analyzing the Potential Therapeutic Mechanism of Huashi Baidu Decoction on Severe COVID-19 through Integrating Network Pharmacological Methods. *J. Tradit. Complement. Med.* **2021**, *11*, doi:10.1016/j.jtcme.2021.01.004.
20. Kumar, S. COVID-19: A Drug Repurposing and Biomarker Identification by Using Comprehensive Gene-Disease Associations through Protein-Protein Interaction Network Analysis. *Preprints* **2020**, doi:10.20944/preprints202003.0440.v1.
21. Nain, Z.; Rana, H.K.; Liò, P.; Islam, S.M.S.; Summers, M.A.; Moni, M.A. Pathogenetic Profiling of COVID-19 and SARS-like Viruses. *Brief. Bioinform.* **2021**, *22*, doi:10.1093/bib/bbaa173.
22. Li, Z.; Yang, L. Underlying Mechanisms and Candidate Drugs for COVID-19 Based on the Connectivity Map Database. *Front. Genet.* **2020**, *11*, doi:10.3389/fgene.2020.558557.
23. Islam, T.; Rahman, M.R.; Aydin, B.; Beklen, H.; Arga, K.Y.; Shahjaman, M. Integrative Transcriptomics Analysis of Lung Epithelial Cells and Identification of Repurposable Drug Candidates for COVID-19. *Eur. J. Pharmacol.* **2020**, *887*, doi:10.1016/j.ejphar.2020.173594.
24. Ge, C.; He, Y. In Silico Prediction of Molecular Targets of Astragaloside IV for Alleviation of COVID-19 Hyperinflammation by Systems Network Pharmacology and Bioinformatic Gene Expression Analysis. *Front. Pharmacol.* **2020**, *11*, doi:10.3389/fphar.2020.556984.
25. Aishwarya, S.; Gunasekaran, K.; Margret, A.A. Computational Gene Expression Profiling in the Exploration of Biomarkers, Non-Coding Functional RNAs and Drug Perturbagens for COVID-19. *J. Biomol. Struct. Dyn.* **2020**, *0*, 1–16, doi:10.1080/07391102.2020.1850360.
26. Tao, Q.; Du, J.; Li, X.; Zeng, J.; Tan, B.; Xu, J.; Lin, W.; Chen, X. lin Network Pharmacology and Molecular Docking Analysis on Molecular Targets and Mechanisms of Huashi Baidu Formula in the Treatment of COVID-19. *Drug Dev. Ind. Pharm.* **2020**, *46*, 1–9, doi:10.1080/03639045.2020.1788070.
27. Beck, B.R.; Shin, B.; Choi, Y.; Park, S.; Kang, K. Predicting Commercially Available Antiviral Drugs That May Act on the Novel Coronavirus (SARS-CoV-2) through a Drug-Target Interaction Deep Learning Model. *Comput. Struct. Biotechnol. J.* **2020**, *18*, doi:10.1016/j.csbj.2020.03.025.
28. Prasad, K.; Khatoon, F.; Rashid, S.; Ali, N.; AlAsmari, A.F.; Ahmed, M.Z.; Alqahtani, A.S.; Alqahtani, M.S.; Kumar, V. Targeting Hub Genes and Pathways of Innate Immune Response in COVID-19: A Network Biology Perspective. *Int. J. Biol. Macromol.* **2020**, *163*, doi:10.1016/j.ijbiomac.2020.06.228.
29. Han, L.; Wei, X.X.; Zheng, Y.J.; Zhang, L.L.; Wang, X.M.; Yang, H.Y.; Ma, X.; Zhao, L.H.; Tong, X.L. Potential Mechanism Prediction of Cold-Damp Plague Formula against COVID-19 via Network Pharmacology Analysis and Molecular Docking. *Chinese Med. (United Kingdom)* **2020**, *15*, doi:10.1186/s13020-020-00360-8.
30. Khan, S.A.; Zia, K.; Ashraf, S.; Uddin, R.; Ul-Haq, Z. Identification of Chymotrypsin-like Protease Inhibitors of SARS-CoV-2 via Integrated Computational Approach. *J. Biomol. Struct. Dyn.* **2021**, *39*, 2607–2616, doi:10.1080/07391102.2020.1751298.
31. Alves, V.M.; Bobrowski, T.; Melo-Filho, C.C.; Korn, D.; Auerbach, S.; Schmitt, C.; Muratov, E.N.;

Tropsha, A. QSAR Modeling of SARS-CoV Mpro Inhibitors Identifies Sufugolix, Cenicriviroc, Proglumetacin, and Other Drugs as Candidates for Repurposing against SARS-CoV-2. *Mol. Inform.* **2021**, *40*, doi:10.1002/minf.202000113.
32. Umesh; Kundu, D.; Selvaraj, C.; Singh, S.K.; Dubey, V.K. Identification of New Anti-NCoV Drug Chemical Compounds from Indian Spices Exploiting SARS-CoV-2 Main Protease as Target. *J. Biomol. Struct. Dyn.* **2021**, *39*, 3428–3434, doi:10.1080/07391102.2020.1763202.
33. Liu, S.; Zheng, Q.; Wang, Z. Potential Covalent Drugs Targeting the Main Protease of the SARS-CoV-2 Coronavirus. *Bioinformatics* **2020**, *36*, doi:10.1093/bioinformatics/btaa224.
34. Jin, Z.; Du, X.; Xu, Y.; Deng, Y.; Liu, M.; Zhao, Y.; Zhang, B.; Li, X.; Zhang, L.; Peng, C.; et al. Structure of Mpro from SARS-CoV-2 and Discovery of Its Inhibitors. *Nature* **2020**, *582*, 289–293, doi:10.1038/s41586-020-2223-y.
35. Feng, Z.; Chen, M.; Xue, Y.; Liang, T.; Chen, H.; Zhou, Y.; Nolin, T.D.; Smith, R.B.; Xie, X.Q. MCCS: A Novel Recognition Pattern-Based Method for Fast Track Discovery of Anti-SARS-CoV-2 Drugs. *Brief. Bioinform.* **2021**, *22*, 946–962, doi:10.1093/bib/bbaa260.
36. Günther, S.; Reinke, P.Y.A.; Fernández-Garciá, Y.; Lieske, J.; Lane, T.J.; Ginn, H.M.; Koua, F.H.M.; Ehrt, C.; Ewert, W.; Oberthuer, D.; et al. X-Ray Screening Identifies Active Site and Allosteric Inhibitors of SARS-CoV-2 Main Protease. *Science (80-.).* **2021**, *372*, doi:10.1126/science.abf7945.
37. Khan, R.J.; Jha, R.K.; Amera, G.M.; Jain, M.; Singh, E.; Pathak, A.; Singh, R.P.; Muthukumaran, J.; Singh, A.K. Targeting SARS-CoV-2: A Systematic Drug Repurposing Approach to Identify Promising Inhibitors against 3C-like Proteinase and 2′-O-Ribose Methyltransferase. *J. Biomol. Struct. Dyn.* **2021**, *39*, 2679–2692, doi:10.1080/07391102.2020.1753577.
38. Raj, R. Analysis of Non-Structural Proteins, NSPs of SARS-CoV-2 as Targets for Computational Drug Designing. *Biochem. Biophys. Reports* **2021**, *25*, 100847, doi:10.1016/j.bbrep.2020.100847.
39. Kuo, C.J.; Chao, T.L.; Kao, H.C.; Tsai, Y.M.; Liu, Y.K.; Wang, L.H.C.; Hsieh, M.C.; Chang, S.Y.; Liang, P.H. Kinetic Characterization and Inhibitor Screening for the Proteases Leading to Identification of Drugs against SARS-CoV-2. *Antimicrob. Agents Chemother.* **2021**, *65*, doi:10.1128/AAC.02577-20.
40. Anand, N.M.; Liya, D.H.; Pradhan, A.K.; Tayal, N.; Bansal, A.; Donakonda, S.; Jainarayanan, A.K. A Comprehensive SARS-CoV-2 Genomic Analysis Identifies Potential Targets for Drug Repurposing. *PLoS One* **2021**, *16*, doi:10.1371/journal.pone.0248553.
41. Cavasotto, C.N.; Di Filippo, J.I. In Silico Drug Repurposing for COVID-19: Targeting SARS-CoV-2 Proteins through Docking and Consensus Ranking. *Mol. Inform.* **2021**, *40*, 1–8, doi:10.1002/minf.202000115.
42. Gil, C.; Ginex, T.; Maestro, I.; Nozal, V.; Barrado-Gil, L.; Cuesta-Geijo, M.Á.; Urquiza, J.; Ramírez, D.; Alonso, C.; Campillo, N.E.; et al. COVID-19: Drug Targets and Potential Treatments. *J. Med. Chem.* **2020**, *63*, 12359–12386, doi:10.1021/acs.jmedchem.0c00606.
43. Guedes, I.A.; Costa, L.S.C.; dos Santos, K.B.; Karl, A.L.M.; Rocha, G.K.; Teixeira, I.M.; Galheigo, M.M.; Medeiros, V.; Krempser, E.; Custódio, F.L.; et al. Drug Design and Repurposing with DockThor-VS Web Server Focusing on SARS-CoV-2 Therapeutic Targets and Their Non-Synonym Variants. *Sci. Rep.* **2021**, *11*, 1–20, doi:10.1038/s41598-021-84700-0.
44. Liang, H.; Zhao, L.; Gong, X.; Hu, M.; Wang, H. Virtual Screening FDA Approved Drugs against Multiple Targets of SARS-CoV-2. *Clin. Transl. Sci.* **2021**, *14*, 1123–1132, doi:10.1111/cts.13007.
45. Rahman, F.; Tabrez, S.; Ali, R.; Alqahtani, A.S.; Ahmed, M.Z.; Rub, A. Molecular Docking Analysis of Rutin Reveals Possible Inhibition of SARS-CoV-2 Vital Proteins. *J. Tradit. Complement. Med.* **2021**, *11*, 173–179, doi:10.1016/j.jtcme.2021.01.006.
46. Murugan, N.A.; Kumar, S.; Jeyakanthan, J.; Srivastava, V. Searching for Target-Specific and

Multi-Targeting Organics for Covid-19 in the Drugbank Database with a Double Scoring Approach. *Sci. Rep.* **2020**, *10*, doi:10.1038/s41598-020-75762-7.
47. Manikyam, H.K.; Joshi, S.K. Whole Genome Analysis and Targeted Drug Discovery Using Computational Methods and High Throughput Screening Tools for Emerged Novel Coronavirus (2019-NCoV). *J. Pharm. drug Res.* **2020**, *3*, 341–361.
48. Wu, C.; Liu, Y.; Yang, Y.; Zhang, P.; Zhong, W.; Wang, Y.; Wang, Q.; Xu, Y.; Li, M.; Li, X.; et al. Analysis of Therapeutic Targets for SARS-CoV-2 and Discovery of Potential Drugs by Computational Methods. *Acta Pharm. Sin. B* **2020**, *10*, 766–788, doi:10.1016/j.apsb.2020.02.008.
49. Abdel-Basset, M.; Hawash, H.; Elhoseny, M.; Chakrabortty, R.K.; Ryan, M. Deeph-DTA: Deep Learning for Predicting Drug-Target Interactions: A Case Study of Covid-19 Drug Repurposing. *IEEE Access* **2020**, *8*, 170433–170451, doi:10.1109/ACCESS.2020.3024238.
50. Mishra, C.B.; Pandey, P.; Sharma, R.D.; Malik, M.Z.; Mongre, R.K.; Lynn, A.M.; Prasad, R.; Jeon, R.; Prakash, A. Identifying the Natural Polyphenol Catechin as a Multi-Targeted Agent against SARS-CoV-2 for the Plausible Therapy of COVID-19: An Integrated Computational Approach. *Brief. Bioinform.* **2021**, *22*, 1346–1360, doi:10.1093/bib/bbaa378.
51. Nelakuditi, B.; Shrivastava, A. Drug Repurposing 57 Well-Known Drugs for Three COVID-19 Targets : Mpro , Spike , RdRp. *Biol. Med. Chem.* **2020**, doi:10.33774/chemrxiv-2021-smfn8.
52. Mhatre, S.; Naik, S.; Patravale, V. A Molecular Docking Study of EGCG and Theaflavin Digallate with the Druggable Targets of SARS-CoV-2. *Comput. Biol. Med.* **2021**, *129*, doi:10.1016/j.compbiomed.2020.104137.
53. Joshi, R.S.; Jagdale, S.S.; Bansode, S.B.; Shankar, S.S.; Tellis, M.B.; Pandya, V.K.; Chugh, A.; Giri, A.P.; Kulkarni, M.J. Discovery of Potential Multi-Target-Directed Ligands by Targeting Host-Specific SARS-CoV-2 Structurally Conserved Main Protease. *J. Biomol. Struct. Dyn.* **2020**, *39*, 1–16, doi:10.1080/07391102.2020.1760137.
54. Shi, A.M.; Guo, R.; Wang, Q.; Zhou, J.R. Screening and Molecular Modeling Evaluation of Food Peptides to Inhibit Key Targets of Covid-19 Virus. *Biomolecules* **2021**, *11*, doi:10.3390/biom11020330.
55. Panda, P.K.; Arul, M.N.; Patel, P.; Verma, S.K.; Luo, W.; Rubahn, H.G.; Mishra, Y.K.; Suar, M.; Ahuja, R. Structure-Based Drug Designing and Immunoinformatics Approach for SARS-CoV-2. *Sci. Adv.* **2020**, *6*, 1–15, doi:10.1126/sciadv.abb8097.
56. Jena, S.; Munusami, P.; Mm, B.; Chanda, K. Computationally Approached Inhibition Potential of Tinospora Cordifolia towards COVID-19 Targets. *VirusDisease* **2021**, *32*, doi:10.1007/s13337-021-00666-7.
57. Duverger, E.; Herlem, G.; Picaud, F. A Potential Solution to Avoid Overdose of Mixed Drugs in the Event of Covid-19: Nanomedicine at the Heart of the Covid-19 Pandemic. *J. Mol. Graph. Model.* **2021**, *104*, doi:10.1016/j.jmgm.2021.107834.
58. He, Xiang, JUNYI WANG, Lei Zhang, Qin Ran, Anying Xiong, Shengbin Liu, Dehong Wu, Bin Niu, Ying Xiong, and G.L. Virtual Screening of Potential AEC2 Inhibitors for COVID-19 from Traditional Chinese Medicine. **2021**, doi:https://doi.org/10.21203/rs.3.rs-145338/v1.
59. de Oliveira, O.V.; Rocha, G.B.; Paluch, A.S.; Costa, L.T. Repurposing Approved Drugs as Inhibitors of SARS-CoV-2 S-Protein from Molecular Modeling and Virtual Screening. *J. Biomol. Struct. Dyn.* **2021**, *39*, doi:10.1080/07391102.2020.1772885.
60. Bardaweel, S.K.; Hajjo, R.; Sabbah, D.A. Sitagliptin: A Potential Drug for the Treatment of COVID-19? *Acta Pharm.* **2021**, *71*, 175–184, doi:10.2478/acph-2021-0013.
61. Kabir, E.R.; Mustafa, N.; Nausheen, N.; Sharif Siam, M.K.; Syed, E.U. Exploring Existing Drugs: Proposing Potential Compounds in the Treatment of COVID-19. *Heliyon* **2021**, *7*, doi:10.1016/j.heliyon.2021.e06284.
62. Bojkova, D.; Klann, K.; Koch, B.; Widera, M.; Krause, D.; Ciesek, S.; Cinatl, J.; Münch, C.

Proteomics of SARS-CoV-2-Infected Host Cells Reveals Therapy Targets. *Nature* **2020**, *583*, 469–472, doi:10.1038/s41586-020-2332-7.
63. Aftab, S.O.; Ghouri, M.Z.; Masood, M.U.; Haider, Z.; Khan, Z.; Ahmad, A.; Munawar, N. Analysis of SARS-CoV-2 RNA-Dependent RNA Polymerase as a Potential Therapeutic Drug Target Using a Computational Approach. *J. Transl. Med.* **2020**, *18*, 1–15, doi:10.1186/s12967-020-02439-0.
64. Elfiky, A.A. SARS-CoV-2 RNA Dependent RNA Polymerase (RdRp) Targeting: An in Silico Perspective. *J. Biomol. Struct. Dyn.* **2021**, *39*, 3204–3212, doi:10.1080/07391102.2020.1761882.
65. Pirzada, R.H.; Haseeb, M.; Batool, M.; Kim, M.S.; Choi, S. Remdesivir and Ledipasvir among the FDA-Approved Antiviral Drugs Have Potential to Inhibit SARS-CoV-2 Replication. *Cells* **2021**, *10*, doi:10.3390/cells10051052.
66. Agrawal, L.; Poullikkas, T.; Eisenhower, S.; Monsanto, C.; Bakku, R.K.; Chen, M.-H.; Kalra, R.S. Viroinformatics-Based Analysis of SARS-CoV-2 Core Proteins for Potential Therapeutic Targets. *Antibodies* **2021**, *10*, 3, doi:10.3390/antib10010003.
67. Ruan, Z.; Liu, C.; Guo, Y.; He, Z.; Huang, X.; Jia, X.; Yang, T. SARS-CoV-2 and SARS-CoV: Virtual Screening of Potential Inhibitors Targeting RNA-Dependent RNA Polymerase Activity (NSP12). *J. Med. Virol.* **2021**, *93*, 389–400, doi:10.1002/jmv.26222.
68. Sun, Y.J.; Velez, G.; Parsons, D.E.; Li, K.; Ortiz, M.E.; Sharma, S.; McCray, P.B.; Bassuk, A.G.; Mahajan, V.B. Structure-Based Phylogeny Identifies Avoralstat as a TMPRSS2 Inhibitor That Prevents SARS-CoV-2 Infection in Mice. *J. Clin. Invest.* **2021**, *131*, doi:10.1172/JCI147973.
69. Cho, T.; Han, H.S.; Jeong, J.; Park, E.M.; Shim, K.S. A Novel Computational Approach for the Discovery of Drug Delivery System Candidates for Covid-19. *Int. J. Mol. Sci.* **2021**, *22*, 1–20, doi:10.3390/ijms22062815.
70. Gao, X.; Qin, B.; Chen, P.; Zhu, K.; Hou, P.; Wojdyla, J.A.; Wang, M.; Cui, S. Crystal Structure of SARS-CoV-2 Papain-like Protease. *Acta Pharm. Sin. B* **2021**, *11*, 237–245, doi:10.1016/j.apsb.2020.08.014.
71. Zhao, Y.; Du, X.; Duan, Y.; Pan, X.; Sun, Y.; You, T.; Han, L.; Jin, Z.; Shang, W.; Yu, J.; et al. High-Throughput Screening Identifies Established Drugs as SARS-CoV-2 PLpro Inhibitors. *Protein Cell* **2021**, doi:10.1007/s13238-021-00836-9.
72. Weglarz-Tomczak, E.; Tomczak, J.M.; Talma, M.; Burda-Grabowska, M.; Giurg, M.; Brul, S. Identification of Ebselen and Its Analogues as Potent Covalent Inhibitors of Papain-like Protease from SARS-CoV-2. *Sci. Rep.* **2021**, *11*, 1–10, doi:10.1038/s41598-021-83229-6.
73. Sinha, S.K.; Shakya, A.; Prasad, S.K.; Singh, S.; Gurav, N.S.; Prasad, R.S.; Gurav, S.S. An In-Silico Evaluation of Different Saikosaponins for Their Potency against SARS-CoV-2 Using NSP15 and Fusion Spike Glycoprotein as Targets. *J. Biomol. Struct. Dyn.* **2021**, *39*, 3244–3255, doi:10.1080/07391102.2020.1762741.
74. Yang, L.; Pei, R. juan; Li, H.; Ma, X. na; Zhou, Y.; Zhu, F. hua; He, P. lan; Tang, W.; Zhang, Y. cheng; Xiong, J.; et al. Identification of SARS-CoV-2 Entry Inhibitors among Already Approved Drugs. *Acta Pharmacol. Sin.* **2021**, *42*, 1347–1353, doi:10.1038/s41401-020-00556-6.
75. Jeon, S.; Ko, M.; Lee, J.; Choi, I.; Byun, S.Y.; Park, S.; Shum, D.; Kim, S. Identification of Antiviral Drug Candidates against SARS-CoV-2 from FDA-Approved Drugs. *Antimicrob. Agents Chemother.* **2020**, *64*, 1–9, doi:10.1128/AAC.00819-20.
76. Kumar, D.; Kumari, K.; Jayaraj, A.; Kumar, V.; Kumar, R.V.; Dass, S.K.; Chandra, R.; Singh, P. Understanding the Binding Affinity of Noscapines with Protease of SARS-CoV-2 for COVID-19 Using MD Simulations at Different Temperatures. *J. Biomol. Struct. Dyn.* **2021**, *39*, 2659–2672, doi:10.1080/07391102.2020.1752310.
77. Liu, C.; Zhu, X.; Lu, Y.; Zhang, X.; Jia, X.; Yang, T. Potential Treatment with Chinese and Western Medicine Targeting NSP14 of SARS-CoV-2. *J. Pharm. Anal.* **2021**, *11*, 272–277,

doi:10.1016/j.jpha.2020.08.002.
78. El Hassab, M.A.; Ibrahim, T.M.; Shoun, A.A.; Al-Rashood, S.T.; Alkahtani, H.M.; Alharbi, A.; Eskandrani, R.O.; Eldehna, W.M. In Silico Identification of Potential SARS COV-2 2′- O - Methyltransferase Inhibitor: Fragment-Based Screening Approach and MM-PBSA Calculations. *RSC Adv.* **2021**, *11*, 16026–16033, doi:10.1039/d1ra01809d.
79. Li, G.; Ruan, S.; Zhao, X.; Liu, Q.; Dou, Y.; Mao, F. Transcriptomic Signatures and Repurposing Drugs for COVID-19 Patients: Findings of Bioinformatics Analyses. *Comput. Struct. Biotechnol. J.* **2021**, *19*, doi:10.1016/j.csbj.2020.11.056.
80. Sun, F.; Mu, C.; Kwok, H.F.; Xu, J.; Wu, Y.; Liu, W.; Sabatier, J.-M.; Annweiler, C.; Li, X.; Cao, Z.; et al. Capivasertib Restricts SARS-CoV-2 Cellular Entry: A Potential Clinical Application for COVID-19. *Int. J. Biol. Sci.* **2021**, *17*, 2348–2355, doi:10.7150/ijbs.57810.
81. Dittmar, M.; Lee, J.S.; Whig, K.; Segrist, E.; Li, M.; Kamalia, B.; Castellana, L.; Ayyanathan, K.; Cardenas-Diaz, F.L.; Morrisey, E.E.; et al. Drug Repurposing Screens Reveal Cell-Type-Specific Entry Pathways and FDA-Approved Drugs Active against SARS-Cov-2. *Cell Rep.* **2021**, *35*, 108959, doi:10.1016/j.celrep.2021.108959.
82. Han, N.; Hwang, W.; Tzelepis, K.; Schmerer, P.; Yankova, E.; MacMahon, M.; Lei, W.; Katritsis, N.M.; Liu, A.; Felgenhauer, U.; et al. Identification of SARS-CoV-2–Induced Pathways Reveals Drug Repurposing Strategies. *Sci. Adv.* **2021**, *7*, 1–15, doi:10.1126/sciadv.abh3032.
83. Li, Y.; Duche, A.; Sayer, M.R.; Roosan, D.; Khalafalla, F.G.; Ostrom, R.S.; Totonchy, J.; Roosan, M.R. SARS-CoV-2 Early Infection Signature Identified Potential Key Infection Mechanisms and Drug Targets. *BMC Genomics* **2021**, *22*, 1–13, doi:10.1186/s12864-021-07433-4.
84. Sauvat, A.; Ciccosanti, F.; Colavita, F.; Di Rienzo, M.; Castilletti, C.; Capobianchi, M.R.; Kepp, O.; Zitvogel, L.; Fimia, G.M.; Piacentini, M.; et al. On-Target versus off-Target Effects of Drugs Inhibiting the Replication of SARS-CoV-2. *Cell Death Dis.* **2020**, *11*, doi:10.1038/s41419-020-02842-x.
85. Krafcikova, P.; Silhan, J.; Nencka, R.; Boura, E. Structural Analysis of the SARS-CoV-2 Methyltransferase Complex Involved in RNA Cap Creation Bound to Sinefungin. *Nat. Commun.* **2020**, *11*, 1–7, doi:10.1038/s41467-020-17495-9.
86. Francis Borgio, J.; Alsuwat, H.S.; Al Otaibi, W.M.; Ibrahim, A.M.; Almandil, N.B.; Al Asoom, L.I.; Salahuddin, M.; Kamaraj, B.; AbdulAzeez, S. State-of-the-Art Tools Unveil Potent Drug Targets amongst Clinically Approved Drugs to Inhibit Helicase in SARS-CoV-2. *Arch. Med. Sci.* **2020**, *16*, 508–518, doi:10.5114/aoms.2020.94567.
87. Díaz, J. SARS-CoV-2 Molecular Network Structure. *Front. Physiol.* **2020**, *11*, 1–8, doi:10.3389/fphys.2020.00870.
88. Gordon, D.E.; Jang, G.M.; Bouhaddou, M.; Xu, J.; Obernier, K.; White, K.M.; O'Meara, M.J.; Rezelj, V. V.; Guo, J.Z.; Swaney, D.L.; et al. A SARS-CoV-2 Protein Interaction Map Reveals Targets for Drug Repurposing. *Nature* **2020**, *583*, doi:10.1038/s41586-020-2286-9.
89. Auwul, M.R.; Rahman, M.R.; Gov, E.; Shahjaman, M.; Moni, M.A. Bioinformatics and Machine Learning Approach Identifies Potential Drug Targets and Pathways in COVID-19. *Brief. Bioinform.* **2021**, doi:10.1093/bib/bbab120.
90. Belyaeva, A.; Cammarata, L.; Radhakrishnan, A.; Squires, C.; Yang, K.D.; Shivashankar, G. V.; Uhler, C. Causal Network Models of SARS-CoV-2 Expression and Aging to Identify Candidates for Drug Repurposing. *Nat. Commun.* **2021**, *12*, doi:10.1038/s41467-021-21056-z.
91. Lee, H.; Park, J.; Im, H.J.; Na, K.J.; Choi, H. Discovery of Potential Imaging and Therapeutic Targets for Severe Inflammation in COVID-19 Patients. *Sci. Rep.* **2021**, *11*, doi:10.1038/s41598-021-93743-2.
92. Desvaux, E.; Hamon, A.; Hubert, S.; Boudjeniba, C.; Chassagnol, B.; Swindle, J.; Aussy, A.; Laigle, L.; Laplume, J.; Soret, P.; et al. Network-Based Repurposing Identifies Anti- Alarmins as Drug Candidates to Control Severe Lung Inflammation in COVID-19. *PLoS One* **2021**, *16*,

doi:10.1371/journal.pone.0254374.
93. O'Donovan, S.M.; Imami, A.; Eby, H.; Henkel, N.D.; Creeden, J.F.; Asah, S.; Zhang, X.; Wu, X.; Alnafisah, R.; Taylor, R.T.; et al. Identification of Candidate Repurposable Drugs to Combat COVID-19 Using a Signature-Based Approach. *Sci. Rep.* **2021**, *11*, doi:10.1038/s41598-021-84044-9.
94. Yee, S.W.; Vora, B.; Oskotsky, T.; Zou, L.; Jakobsen, S.; Enogieru, O.J.; Koleske, M.L.; Kosti, I.; Rödin, M.; Sirota, M.; et al. Drugs in COVID-19 Clinical Trials: Predicting Transporter-Mediated Drug-Drug Interactions Using In Vitro Assays and Real-World Data. *Clin. Pharmacol. Ther.* **2021**, *110*, doi:10.1002/cpt.2236.
95. Zhou, Y.; Hou, Y.; Shen, J.; Huang, Y.; Martin, W.; Cheng, F. Network-Based Drug Repurposing for Novel Coronavirus 2019-NCoV/SARS-CoV-2. *Cell Discov.* **2020**, *6*, doi:10.1038/s41421-020-0153-3.
96. Alam, T.; Lipovich, L. Mircovid-19: Potential Targets of Human Mirnas in Sars-Cov-2 for Rna-Based Drug Discovery. *Non-coding RNA* **2021**, *7*, doi:10.3390/NCRNA7010018.
97. Alanazi, K.M.; Farah, M.A.; Hor, Y.Y. Multi-Targeted Approaches and Drug Repurposing Reveal Possible SARS-CoV-2 Inhibitors. *Vaccines* **2022**, *10*, doi:10.3390/vaccines10010024.
98. Jose, S.; Gupta, M.; Sharma, U.; Quintero-Saumeth, J.; Dwivedi, M. Potential of Phytocompounds from Brassica Oleracea Targeting S2-Domain of SARS-CoV-2 Spike Glycoproteins: Structural and Molecular Insights. *J. Mol. Struct.* **2022**, *1254*, doi:10.1016/j.molstruc.2022.132369.
